# Cell Uptake and Biocompatibility of Nanoparticles Prepared from Poly(benzyl malate) (Co)polymers Obtained through Chemical and Enzymatic Polymerization in Human HepaRG Cells and Primary Macrophages

**DOI:** 10.3390/polym10111244

**Published:** 2018-11-10

**Authors:** Hubert Casajus, Saad Saba, Manuel Vlach, Elise Vène, Catherine Ribault, Sylvain Tranchimand, Caroline Nugier-Chauvin, Eric Dubreucq, Pascal Loyer, Sandrine Cammas-Marion, Nicolas Lepareur

**Affiliations:** 1 Ecole Nationale Supérieure de Chimie de Rennes, Univ Rennes, CNRS, ISCR, UMR 6226, F-35000 Rennes, France; casajus.hubert@gmail.com (H.C.); sylvain.tranchimand@ensc-rennes.fr (S.T.); caroline.nugier@ensc-rennes.fr (C.N.-C.); 2 Univ Rennes, INSERM, INRA, Institut NUMECAN (Nutrition Metabolisms and Cancer) UMR_A 1341, UMR_S 1241, F-35000 Rennes, France; saad.saba@etudiant.univ-rennes1.fr (S.S.); manuel.vlach@univ-rennes1.fr (M.V.); elise.vene@univ-rennes1.fr (E.V.); catherine.ribault@univ-rennes1.fr (C.R.); 3 Montpellier SupAgro, INRA, CIRAD, Univ Montpellier, UMR 1208 IATE, F-34060 Montpellier, France; eric.dubreucq@supagro.fr; 4 Comprehensive Cancer Center Eugène Marquis, F-35000 Rennes, France

**Keywords:** enzymatic polymerization, chemical polymerization, poly(benzyl malate), biocompatible nanoparticles, cell uptake, cytotoxicity, HepaRG cells, human macrophages

## Abstract

The design of drug-loaded nanoparticles (NPs) appears to be a suitable strategy for the prolonged plasma concentration of therapeutic payloads, higher bioavailability, and the reduction of side effects compared with classical chemotherapies. In most cases, NPs are prepared from (co)polymers obtained through chemical polymerization. However, procedures have been developed to synthesize some polymers via enzymatic polymerization in the absence of chemical initiators. The aim of this work was to compare the acute in vitro cytotoxicities and cell uptake of NPs prepared from poly(benzyl malate) (PMLABe) synthesized by chemical and enzymatic polymerization. Herein, we report the synthesis and characterization of eight PMLABe-based polymers. Corresponding NPs were produced, their cytotoxicity was studied in hepatoma HepaRG cells, and their uptake by primary macrophages and HepaRG cells was measured. In vitro cell viability evidenced a mild toxicity of the NPs only at high concentrations/densities of NPs in culture media. These data did not evidence a higher biocompatibility of the NPs prepared from enzymatic polymerization, and further demonstrated that chemical polymerization and the nanoprecipitation procedure led to biocompatible PMLABe-based NPs. In contrast, NPs produced from enzymatically synthesized polymers were more efficiently internalized than NPs produced from chemically synthesized polymers. The efficient uptake, combined with low cytotoxicity, indicate that PMLABe-based NPs are suitable nanovectors for drug delivery, deserving further evaluation in vivo to target either hepatocytes or resident liver macrophages.

## 1. Introduction

Biocompatible polymeric nanoparticles combined with the design of more specific therapeutic molecules are developed toward the goal of reducing the drug amounts administrated to patients, simplifying protocols of administration, and overcoming side effects. This research is based on the “Magic Bullet” concept first coined by Paul Ehrlich [1] at the beginning of the 20th century, which was a concept that inspired generations of scientists in the design of more efficient therapeutic molecules and vectorized therapies. Since the end of the 20th century, progress in this area has been significant, and has led to the design of nanovectors approved by the United States (US) Food and Drug Administration (FDA), or in pre-clinical or clinical phases [2,3,4,5,6,7].

The design of nanovectors for clinical applications implies very strict specifications. They must: (i) be biocompatible and/or (bio)degradable; (ii) be easy to produce in Good Manufacturing Practice (GMP) grade for clinical use; (iii) allow the encapsulation of a significant amount of active molecules such as drugs, genes, or peptides [8]; (iv) lead to the controlled release of active molecules at the targeted sites (cells, tissues, and/or organs) through the presence of targeting agents (antibodies, peptides, vitamins) [9,10,11]; and (v) have controlled surface characteristics to minimize their recognition by the immune system. These nanovectors can take the form of active molecule–polymers conjugates [12], nanoparticles [13], micelles [14], or polymersomes [15].

Aliphatic polyesters are produced for multiple industrial use such as the plastic bag industry instead of poly(ethylene), which is a non-biodegradable polymer derived directly from petrochemicals [16]. Their remarkable properties of biodegradability and biocompatibility make them also suitable for biomedical and pharmacological applications, including the preparation of nanoparticles (NPs) for drug delivery [11,17].

Such aliphatic polyesters are usually synthesized by the polymerization of the corresponding monomer(s) using chemically-based initiators [18]. On the other hand, some of these polyesters can be extracted from fungi, such as for instance the natural poly(malic acid) extracted from *Physarum polycephalum* [19], or may be prepared by biotechnological means using a large-scale culture of bacteria as for polyhydroxyalkanoates or (PHAs) [20]. The advantage of these biotechnological pathways relies mainly in the use of so-called “green” synthetic processes. However, it is difficult, if not impossible, to control the molar masses of the polymers, and only chiral polymers are obtained when the repeating unit of the block (co)polymers has one or more asymmetric carbon(s). Therefore, the so-called conventional “chemical” methods of synthesis cannot, at present, be totally ruled out. However, authors have pointed out the question of whether the initiators and/or catalysts that are used during these polymerization reactions might induce cytotoxicities when the corresponding polymers are in contact with cells in living organisms, even if initiators and catalysts are present only as traces in the final material [21]. Among the aliphatic polyesters’ family, poly(malic acid) (PMLA) and its derivatives have raised a growing interest in the biomedical field because of their properties of biocompatibility and biodegradability [22,23] and potential applications in drug delivery. In addition to these remarkable properties, a whole family of PMLA derivatives can be obtained by modifying the synthesis of the monomers and the reactions of the homopolymerization and copolymerization of these monomers [24,25]. We and others have recently developed PMLA-based nanovectors, taking advantage of the exceptional properties of such polymers [22,24,26,27,28]. The first nanovector is a polymer-drug conjugate constituted of a PMLA whose lateral carboxylic acids were modified to graft different molecules of interest: doxorubicin as a drug, poly(ethylene glycol) to improve the hydrophilic properties of the conjugate, and N-acetyl galactosamine as a targeting agent [29]. Furthermore, we also prepared NPs from either hydrophobic poly(benzyl malate) (PMLABe) or amphiphilic poly(ethylene glycol)-*block*-PMLABe (PEG-*b*-PMLABe) having or not having the biotin as a targeting agent at the free end of the PEG block [22,23,30,31].

This family of polyesters is obtained by the anionic ring-opening polymerization (AROP) of alkyl malolactonates (monomers) in the presence of tetraethylammonium carboxylate salts, and more particularly tetraethylammonium benzoate [32]. It is important to note that this last initiator is a powerful neurotoxin [33]. Therefore, the use of biocatalysts, such as lipases, is an interesting alternative to avoid the presence of traces of toxic chemical initiators in polymeric materials. Lipases are enzymes belonging to the family of hydrolases, which is class 3 in the EC nomenclature (Enzyme Commission numbers). More specifically, they are serine hydrolases that have the particularity of being active in the presence of an interface, called activation, between an aqueous phase (water or buffer) and an organic phase (hydrophobic substrate and/or organic solvent). Present in plants as well as in insects, animals, and microorganisms, lipases play a key role in the degradation and synthesis of lipids [34]. The physiological reaction catalyzed by lipases is the hydrolysis of mono, di, and triglycerides. However, they are also capable of synthesizing esters, either by esterification or transesterification, when the activity of the water is low. This property is one of the main reasons for the use of this class of enzymes, along with their high selectivity during hydrolysis [34]. More than 100 three-dimensional structures are deposited on the Protein Data Bank (PDB), which has made it possible to highlight the highly conserved structural elements specific to lipases [34]. The canonical mechanism of hydrolysis of ester functions involves the three amino acids of the catalytic triad, namely, aspartate, histidine, and serine [35].

These enzymes have been studied as potential catalysts in the ring-opening polymerization of unsubstituted lactones such as ε-caprolactones [36]. The mechanism of this enzymatic catalyzed ring opening polymerization of unsubstituted lactones has been extensively studied and described in the literature: it occurs through the canonical mechanism described for the hydrolysis of esters’ functions [37,38,39]. However, despite the interest of such enzymatic polymerization, several technological barriers still need to be overcome to completely replace the chemical initiators/catalysts. Indeed, enzymatic polymerizations are, to date, less controlled than chemical ones [40,41,42]. Nevertheless, we recently set up conditions to synthesize well-defined poly(benzyl malate) (PMLABe) derivatives of high molar masses through the enzymatic polymerization of benzyl malolactonate (MLABe) in the presence of pancreatic porcine lipase (PPL) [43].

Taking advantage of the availability of (co)polymers synthesized by polymerization in the presence of chemical initiators and enzymatic polymerization, the aim of this work was to compare the acute in vitro cytotoxicity of NPs prepared from PMLABe derivatives synthesized either by chemical (PMLABe chemicals) or enzymatic (PMLABe enzymatic) polymerization and their cell uptake. For that purpose, we have synthesized and characterized a series of eight PMLABe-based polymers, formulated the corresponding NPs, and studied their cytotoxicity in progenitor and differentiated hepatoma HepaRG cells, and measured their uptake by both primary macrophages and HepaRG cells.

## 2. Materials and Methods

### 2.1. Materials and Apparatus

All of the chemicals were used as received. α-methoxy,ω-carboxylic acid PEG_45_ ($\bar{M}w$ = 2000 g/mol, n = 45) and α-hydroxyl,ω-methoxy-PEG_17_ ($\bar{M}w$ = 736 g/mol, n = 17) were purchased from PEG Iris Biotech. Porcine pancreatic lipase (PPL) was purchased from Sigma-Aldrich Co. (Saint Louis, MO, USA).

**Nuclear magnetic resonance spectroscopy**: The standard temperature was adjusted to 298K. NMR spectra were recorded on a Bruker Avance III 400 spectrometer (Billerica, MA, USA) operating at 400.13 MHz for ^1^H, equipped with a BBFO probe with a Z-gradient coil and a GREAT 1/10 gradient unit. The zg30 Bruker pulse program was used for 1D ^1^H NMR, with a TD of 64 k, a relaxation delay d1 = 2 s, and eight scans. The spectrum width was set to 18 ppm. Fourier transform of the acquired FID was performed without any apodization in most cases.

**Infrared spectroscopy:** Fourier transform infrared (FT-IR) spectra were recorded on an Avatar 320FT-IR Thermo Nicolet spectrometer (ThermoFischer, Waltham, MA, USA) between 500–4000 cm^−1^ by direct measurement.

**Size exclusion chromatography:** Mass average molar mass ($\bar{M}w$) and dispersity (Đ = $\bar{M}w$/$\bar{M}n$) values were measured by size exclusion chromatography (SEC) in THF at 40 °C (flow rate = 1.0 mL/min) on a GPC2502 Viscotek apparatus (Malvern Instruments, Malvern, UK) equipped with a refractive index detector Viscotek VE 3580 RI, a guard column Viscotek TGuard, Org 10 mm × 4.6 mm, a LT5000L gel column (for samples soluble in organic medium) 300 mm × 7.8 mm, and a GPC/SEC OmniSEC Software. The polymer samples were dissolved in THF (2 mg/mL). All of the elution curves were calibrated with polystyrene standards.

**Polarimetry**: Optical rotations were measured using a Perkin-Elmer 341 polarimeter (Waltham, MA, USA) with a sodium lamp (wavelength = 589 nm). Samples were dissolved in chloroform (concentration around 10 mg/mL) and transferred into the polarimeter microcell (length = 1 dm). Measures were performed at room temperature.

**Dynamic light scattering**: The size and polydispersity index (PDI) of the formulations were measured by dynamic light scattering using a Zetasizer Nano-ZR90 (Malvern Instruments, Malvern, UK) apparatus at 25 °C, an He-Ne laser at 633 nm, and a detection angle of 90°. Data were processed by the Zetasizer Software v7.11 (Malvern Instruments, Malvern, UK).

**Flow cytometry**: Cell monolayers were detached with trypsin and analyzed by flow cytometry (Becton Dickinson le LSRFortessa™ X-20, Franklin Lakes, NJ, USA), using the cytometry core facility of the Biology and Health Federative research structure Biosit, Rennes, France to quantify the fluorescence emitted by the DiIC18 (7) (1,1″-dioctadecyl-3,3,3″,3″-tetramethylindotricarbocyanine iodide) (DiR)-loaded NPs within cells. Cytometry data were analyzed using DIVA software (Becton Dikinson, Franklin Lakes, NJ, USA). 

### 2.2. Synthesis of Monomers and Polymers

**Synthesis of racemic and optically active MLABe:** RS, R, and S-MLABe were synthesized as previously described [32]. Briefly, dl, l, or d-aspartic acid (1 eq) was reacted with sodium bromide (5.5 eq) and sodium nitrite (1.2 eq) in 2N H_2_SO_4_ leading, after recrystallization in acetonitrile, to the corresponding pure RS, S, or R-bromosuccinic acid. The racemic or optically active bromosuccinic acid was dissolved in anhydrous THF and reacted with trifluoroacetic anhydride (1.3 eq), leading to the corresponding racemic or optically active bromosuccinic anhydride, which was immediately reacted with benzyl alcohol (1 eq) to obtain the corresponding mixture of racemic or optically monoesters. The pH of this mixture of racemic or optically active monoesters was increased to 7.2 by addition of 2N NaOH; the resulting aqueous solution was mixed with dichloromethane, and the biphasic medium was heated at 45 °C. After purification (column chromatography and distillation under vacuum), the pure RS, R, or S-MLABe was obtained and characterized by FT-IR and ^1^H NMR (Figure SI).

RS-MLABe (Figures SI 1.1 and SI 2.1):

*FT-IR (ν, cm^−1^): 1840 (C=O, lactone) 1750 (C=O, ester)*

*^1^H NMR (400 MHz, Acetone-d_6_), δ (ppm): 7.47–7.28 (m, 5H), 5.27 (s, 2H), 5.11 (dd, J = 6.7, 4.4 Hz, 1H), 4.00–3.60 (dd, J = 16.5, 6.7 Hz, 1H), 3.72 (dd, J = 16.5, 4.4 Hz, 1H).*

S-MLABe (Figures SI 1.2 and 2.2):

*FT-IR (ν, cm^−1^): 1840 (C=O, lactone) 1750 (C=O, ester)*

*^1^H NMR (400 MHz, Acetone-d_6_), δ (ppm): 7.47–7.28 (m, 5H), 5.27 (s, 2H), 5.11 (dd, J = 6.7, 4.4 Hz, 1H), 4.00–3.60 (dd, J = 16.5, 6.7 Hz, 1H), 3.72 (dd, J = 16.5, 4.4 Hz, 1H).*

R-MLABe (Figures SI 1.3 and 2.3):

*FT-IR (ν, cm^−1^): 1840 (C=O, lactone) 1750 (C=O, ester)*

*^1^H NMR (400 MHz, Acetone-d_6_), δ (ppm): 7.47–7.28 (m, 5H), 5.27 (s, 2H), 5.11 (dd, J = 6.7, 4.4 Hz, 1H), 4.00–3.60 (dd, J = 16.5, 6.7 Hz, 1H), 3.72 (dd, J = 16.5, 4.4 Hz, 1H).*

**Chemical polymerization of RS and R-MLABe:** The RS-PMLABe (P1) and S-PMLABe (P2) were synthesized as previously described [32] by anionic ring-opening polymerization (AROP) of the RS-MLABe and R-MLABe, respectively, in presence of tetraethylammonium benzoate as initiator. The theoretical molar mass (30,000 g/mol) was fixed by the ratio monomer/initiator. Both polymers were purified by precipitation into ethanol. After drying under vacuum, the RS-PMLABe and S-PMLABe were analyzed by ^1^H NMR, SEC, and polarimetry.

*RS-PMLABe (P1): ^1^H NMR (400 MHz, Acetone-d6) δ(ppm): 7.40–7.25 (m, 5nH), 5.55 (m, 1nH), 5.16 (m, 2nH), 3.24–2.67 (m, 2nH)—Figure SI 3.1. SEC (THF, 40 °C, polystyrene standards):* $\bar{M}w$ *= 14,850 g/mol, Đ = 1.60. [α]_D_ = 0;*

*S-PMLABe (P2): ^1^H NMR (400 MHz, Acetone-d6) δ(ppm): 7.40–7.25 (m, 5nH), 5.55 (m, 1nH), 5.16 (m, 2nH), 3.24–2.67 (m, 2nH)—Figure SI 3.2. SEC (THF, 40 °C, polystyrene standards): impossible to measure in THF. [α]_D_ = −10.7.*

The PEG_45_-*b*-PMLABe_73_ (P3) was synthesized as previously described [23] through the AROP of RS-MLABe in presence of α-methoxy,ω-carboxylate PEG_45_ tetraethylammonium salt as initiator, in anhydrous THF. In this case also, the PMLABe molar mass was set by the ratio monomer/initiator, and selected to be 15,000 g/mol. The resulting block copolymer was purified by precipitation in ethanol. After drying under vacuum, the pure block copolymer was analyzed by ^1^H NMR and SEC.

PEG_45_-b-PMLABe_73_ (P3): ^1^H NMR (400 MHz; Acetone-d6), δ (ppm): 7.40–7.20(m, 5nH), 5.60–5.49 (m, 1nH), 5.40 (m, 2nH), 3.60 (m, 4mH (m = 45)), 3.10–2.80 (m, 2nH)—Figure SI 3.3. *M*_NMR_ = 15,040 g/mol for the PMLABe block (n = 73). SEC (THF, 40 °C, polystyrene standards): $\bar{M}w$ = 9600 g/mol, Đ = 1.30.

**Enzymatic polymerization of RS, R, and S-MLABe:** The RS-PMLABe P4 was synthesized as described previously [43] by ring-opening polymerization of the RS-MLABe in the presence of porcine pancreatic lipase (PPL) without toluene. Experiments were realized in a 24-multi reactor Büchi Syncore Line (Flawil, Switzerland). In a 30-mL tube, PPL (8.4 mg) was mixed with pure RS-MLABe (336 mg) and Tris/HCl buffer (125 µL, pH = 7, 105 mM). The mixture was stirred at 390 rpm, and the temperature was controlled at 60 °C. After 72 h, reaction was stopped by the addition of 3 mL of THF and 3 mL of chloroform. The mixture was then transferred into a separatory funnel. Separations were realized by adding 15 mL of distilled water and 15 mL of chloroform. Organic phases were dried over MgSO_4_ and filtered. Finally, solvents were evaporated under reduced pressure. The RS-PMLABe P4 was dissolved in chloroform and then precipitated in a large excess of cold diethyl ether. After elimination of the supernatant, the polymer was dried under vacuum and analyzed by ^1^H NMR, SEC, and polarimetry.

*RS-PMLABe (P4): ^1^H NMR (400 MHz, CDCl_3_) δ(ppm): 7.25 (m, 5nH), 5.49 (m, 1nH), 5.08 (m, 2nH), 2.88 (m, 2nH)—Figure SI 3.4. SEC (THF, 40 °C, polystyrene standards):* $\bar{M}w$ *= 12,250 g/mol, Đ = 1.4. [α]_D_ = 0.*

The RS-PMLABe P5, the S-PMLABe P6, and the R-PMLABe P7 were obtained as described previously [43] by ring-opening polymerization of the RS-MLABe, R-MLABe, and S-MLABe, respectively, in the presence of porcine pancreatic lipase (PPL) with toluene. Experiments were realized in a 24-multi reactor Büchi Syncore Line. In a 30-mL tube, PPL (8.4 mg) was mixed with RS-MLABe, R-MLABe, or S-MLABe (338 mg), Tris/HCl (333 µL, pH = 7, 105 mM), and toluene (667 µL). The final volume was 1 mL. The reaction mixtures were stirred at 390 rpm, and the temperature was maintained at 60 °C. After 72 h, reaction was stopped by the addition of 3 mL of THF and 3 mL of chloroform. The mixtures were then transferred into a separatory funnel. Separations were realized by adding 15 mL of distilled water and 15 mL of chloroform. Organic phases were dried over MgSO_4_ and filtered. Finally, solvents were evaporated under reduced pressure. The RS-PMLABe P5, S-PMLABe P6, and R-PMLABe P7 were dissolved in chloroform, and then precipitated in a large excess of cold diethyl ether. After elimination of the supernatant, polymers were dried under vacuum and analyzed by ^1^H NMR, SEC, and polarimetry.

*RS-PMLABe (P5): ^1^H NMR (400 MHz, CDCl_3_) δ(ppm): 7.25 (m, 5nH), 5.49 (m, 1nH), 5.08 (m, 2nH), 2.88 (m, 2nH)—Figure SI 3.5. SEC (THF, 40 °C, Standards Polystyrene):* $\bar{M}w$ *= 3850 g/mol, Đ = 1.5. [α]_D_ = −3.*

*S-PMLABe (P6): ^1^H NMR (400 MHz, CDCl_3_) δ(ppm): 7.25 (m, 5nH), 5.49 (m, 1nH), 5.08 (m, 2nH), 2.88 (m, 2nH)—Figure SI 3.6. SEC (THF, 40 °C, polystyrene standards):* $\bar{M}w$ *= 2000 g/mol, Đ = 1.50. [α]_D_ = −11.*

*R-PMLABe (P7): ^1^H NMR (400 MHz, CDCl_3_) δ(ppm): 7.25 (m, 5nH), 5.49 (m, 1nH), 5.08 (m, 2nH), 2.88 (m, 2nH)—Figure SI 3.7. SEC (THF, 40 °C, polystyrene standards):* $\bar{M}w$ *= 2300 g/mol, Đ = 1.40. [α]_D_ = +18.*

The PEG_17_-b-PMLABe_47_ (P8) was synthesized by enzymatic ring opening polymerization (ROP) of RS-MLABe in the presence of α-methoxy-ω-hydroxy-PEG_17_ and PPL as the catalyst. Before the reaction, the PPL and the α-methoxy-ω-hydroxy-PEG_17_ (*M_w_* = 736 g/mol) were lyophilized for 12 h. In a 30-mL tube, PPL (12.5 mg) was mixed with 427 mg (2.07 mmol, 1eq.) of RS-MLABe, 77 mg (0.1 mmol, 0.05 eq) of α-methoxy-ω-hydroxy-PEG_17_, and anhydrous toluene (630 µL). The reaction was performed at 60 °C at 390 rpm during 72 h. The reaction was stopped by the addition of 5 mL of chloroform. Then, the mixture was transferred in Eppendorf tubes and centrifuged for 3 min at 14,000 rpm. Supernatants were filtered, and the chloroform was evaporated under reduced pressure. Polymer P8 was dissolved in chloroform, and then precipitated in a large excess of cold diethyl ether. After elimination of the supernatant, polymers were dried under reduced pressure at room temperature. The PEG_17_-*b*-PMLABe_47_ (P8) was analyzed by ^1^H NMR (Figure SI) and SEC.

*PEG_17_-b-PMLABe_47_ (P8): ^1^H NMR (400 MHz, Acetone-d6) δ (ppm): 7.57–7.07 (m, 5nH), 5.55 (m, 1nH), 5.16 (m, 2nH), 3.60 (m, 4mH), 3.24–2.67 (m, 2nH).—Figure SI 3.8. SEC (THF, 40 °C, 1 mL/min, polystyrene standards):* $\bar{M}w$ *= 9650 g/mol Đ = 1.50.*

### 2.3. Formulation of PMLABe-Based Nanoparticles

**Formulation of NPs for the cell uptake assays:** NPs were loaded with DiR (DiIC18 (7) (1,1″-dioctadecyl-3,3,3″,3″-tetramethylindotricarbocyanine iodide)). DiR is a hydrophobic carbocyanine-type probe emitting in the near infrared (780 nm). Five mg of the selected polymer were dissolved in 100 µL of DMF, and then 50 µL of DiR (1 mg/mL in dimethylformamide (DMF), corresponding to 1 wt % with the dissolved polymer) was added. This solution was rapidly added into 1 mL of distilled water under vigorous stirring. The nanoparticles formed instantly because of the hydrophobic or amphiphilic nature of the (co)polymers. The suspension was left under stirring for 1 h. Then, DiR-loaded NPs were purified using Sephadex steric exclusion columns (PD-10 column) to eliminate free DiR. The concentration of loaded DiR was measured by UV at 670 nm in a solution prepared by mixing 80 µL of the DiR-loaded nanoparticles with 320 µL of DMF, and using a calibration curve of DiR solution in a mixture of DMF/H_2_O (80/20). The absorbance of the solutions was measured using a Microplate Spectrophotometer powerwase XS/XS2 (Biotek, Winooski, VT, USA), equipped with Gen5 Data Analysis Software (Biotek, Winooski, VT, USA). The NPs were characterized by dynamic light scattering (DLS) in water at room temperature with a concentration in polymer of 2.5 mg/mL.

**Formulation of NPs for the acute toxicity:** NPs were prepared by the nanoprecipitation method as described described [44,45]. Five mg of the selected polymer were dissolved in five mL of 1,4-dioxane. This solution was rapidly added into two mL of distilled water under vigorous stirring. The suspension was left under stirring during 15 min, and the 1,4-dioxane was evaporated under reduced pressure at 40 °C. The final polymer concentration was 2.5 mg/mL. The NPs were characterized by dynamic light scattering (DLS) in water at room temperature with a concentration in polymer of 2.5 mg/mL.

### 2.4. In Vitro Assays

**HepaRG culture:** For all of the studies, proliferating progenitors HepaRG cells were seeded at a density of 3.10^4^ cells/cm^2^ and cultured as previously described [46], in William’s E medium (Lonza, Basel, Switzerland) supplemented with 2 mM of glutamine (Gibco, ThermoFischer, Waltham, MA, USA), 5 mg/L of insulin (Sigma, Saint Louis, MO, USA), 10^−2^ mM of hydrocortisone hemisuccinate, and 10% of fetal calf serum (FCS) (Lonza, Basel, Switzerland). HepaRG cells were cultured in William’s E medium supplemented with 10% of FCS, 50 units/mL of penicillin, 50 µg/mL of streptomycin, 5 µg/mL of insulin, 2 mM of l-glutamine, and 50 µM of hydrocortisone hemisuccinate.

**Primary macrophage culture:** Peripheral blood mononuclear cells were purified from human buffy coat (Etablissement Français du Sang, Rennes, France) by differential centrifugation on UNI-SEP maxi U10 (Novamed, Jerusalem, Israel). The experiments were performed in compliance with the French legislation on blood donation and blood products’ use and safety. Monocytes from healthy donors were enriched using a human CD14 separation kit (Microbeads; Miltenyi Biotec, Bergisch Gladbach, Germany), plated at a density of 0.5 × 10^6^ cells per well in 24-well plates, and cultured at 37 °C with 5% humidified CO_2_ in RPMI 1640 medium supplemented with 100 IU/mL of penicillin, 100 mg/mL of streptomycin, 2 mM of l-glutamine, and 10% FCS BioWhittaker^®^. Macrophages were obtained after differentiation from monocytes by incubation with 50 ng/mL of rhGM-CSF in RPMI 1640 medium for seven days, as previously described [47].

**Cytotoxicity assays:** The cytotoxicity was assessed using the human HepaRG hepatoma cell line and the Thiazolyl Blue Tetrazolium Bromide (MTT) assay. Briefly, cells were incubated with MTT (0.5 mg/mL) for 1 h at 37 °C. The formed crystals were dissolved in DMSO at room temperature for 10 min, and the absorbance was read at 490 nm with a microplate reader. The MTT values reflecting the number of viable cells were expressed in percentage relative to the absorbance determined in control cultures. The cells were exposed to the eight NPs at molar concentrations in polymers ranging from 0.5 μM to 20 μM, corresponding to different mass concentrations, since the polymers exhibit different molar masses. The polymer masses in grams per liter (L) were the following: NPs 1: 15 × 10^−3^ to 0.6 g/L; NPs 2: 15 × 10^−3^ to 0.6 g/L; NPs 3: 7.5 × 10^−3^ to 0.3 g/L; NPs 4: 6.125 × 10^−3^ to 0.245 g/L; NPs 5: 1.925 × 10^−3^ to 0.077 g/L; NPs 6: 10^−3^ to 0.04 g/L; NPs 7: 1.15 × 10^−3^ to 0.046 g/L; NPs 8: 4.8 × 10^−3^ to 0.193 g/L. The same range of molar concentrations in polymers was chosen, since the NPs had relatively similar diameters, indicating that the same amounts of masses of polymers would produce almost nearly identical numbers of NPs. The 5 mg of polymers that was used to prepare NPs (2 mL suspension of water) produced the following numbers of NPs: NPs 1 = 9.9 × 10^11^, NPs 2 = 1.5 × 10^12^, NPs 3 = 1.3 × 10^13^, NPs 4 = 9.2 × 10^11^, NPs 5 = 3.8 × 10^12^, NPs 6 = 2.4 × 10^12^, NPs 7 = 3.3 × 10^12^, and NPs 8 = 1.2 × 10^12^.

**Cell uptake assays:** For the cell uptake assay of NPs, human macrophages and HepaRG cells were plated in 24-well plates. The culture medium of macrophages and HepaRG cells was renewed, and PMLABe-based NPs loaded with DiR were added to the wells for 24 h. After incubation, culture media were discarded, and the cell monolayers were washed once with Phosphate Buffer Saline (PBS); then, they were detached with trypsin and analyzed by flow cytometry. Dot plots of forward scatter (FSC: *x* axis, size of events) and side scatter (SSC: *y* axis, structure of events) allowed to gate the viable cells prior to detecting the fluorescence emitted by DiR-loaded NPs using the BV786-1A channel. Two parameters were analyzed: the percentage of positive cells that internalized NPs and the intensity of fluorescence (mean of fluorescence) reflecting the accumulation of fluorescent NPs within the cells.

**Statistical analyses**: Statistical analyses were performed using a one-way Anova followed by the Kruskal–Wallis post-test or Dunn’s multiple comparison tests. Statistically significant variations after treatment were compared with controls using Student’s *t*-test with Excel software. * *p* < 0.05; ** *p* < 0.01.

## 3. Results and Discussion

### 3.1. Synthesis and Characterization of the Monomers.

**Scheme 1:**
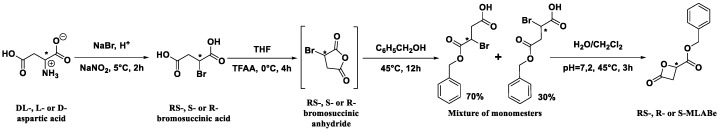
Synthesis of poly(benzyl malate) (MLABe).

The three β-substituted β-lactones involved in this study were synthesized in four steps starting from dl, l, or d-aspartic acid, as described previously (Scheme 1) [32]. 

As shown by the results given in the Experimental part and in the Supplementary Information (Figure SI), the characteristics (yields and ^1^H NMR and FT-IR spectra) of the synthesized RS, S, and R-MLABe were identical to those given in the literature [32]. The l-aspartic acid leads to the R-MLABe, while the d-aspartic acid leads to the S-MLABe. Indeed, the last step of the MLABe’s synthesis occurs through an intramolecular nucleophilic substitution of second-order (S_N_2) reaction [10]. We thus obtained pure racemic and optically active benzyl malolactonates, which can be therefore polymerized in the presence of either chemical or enzymatic initiators.

### 3.2. Synthesis and Characterization of the Polymers

**Scheme 2:**
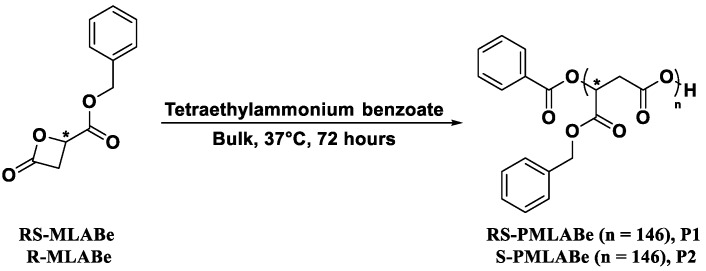
Synthesis of the RS-PMLABe (P1) and S-PMLABe (P2).

Chemical polymerization: Two lactones, the RS and the R-MLABe, were polymerized by anionic ring-opening polymerization (AROP) in the presence of tetraethylammonium benzoate as the initiator, leading to the expected RS- and S-PMLABe, respectively (Scheme 2) [32].

The ring-opening of the monomer occurs via an O-alkyl bond cleavage, and thus with an inversion of configuration of the asymmetric carbon. Therefore, the R-MLABe leads to the S-PMLABe. Due to a fast initiation step, the molar mass of the resulting polymer is controlled by the monomer versus initiator ratio. Within this study, we chose to synthesize RS and S-PMLABe with a theoretical molar mass of 30,000 g/mol. The polymerization’s reaction was followed by FT-IR and stopped when the band characteristic of the lactone ring at 1850 cm^−1^ totally disappeared from the FT-IR spectrum. After reaction, the obtained polymers were purified by precipitation in ethanol and characterized by ^1^H NMR (structure—Figures SI 3.1 and 3.2), SEC (molar mass and dispersity) and polarimetry (Table 1). The SEC analysis on the S-PMLABe was not possible as a result of the insolubility of this polymer into THF.

The synthesized RS-PMLABe (P1) and S-PMLABe (P2) have the expected characteristics and were further used for preparing the corresponding NPs.

**Scheme 3:**
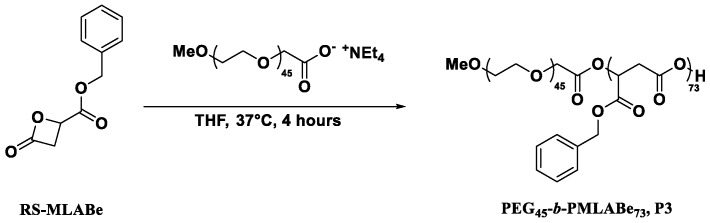
Synthesis of the poly(ethylene glycol)-*block*-PMLABe (PEG_45_-b-PMLABe_73_) (P3).

Besides, we synthesized an amphiphilic block copolymer composed by a hydrophilic block of poly(ethylene glycol) (PEG) and a hydrophobic block of PMLABe by the AROP of the RS-MLABe in the presence of α-methoxy,ω-carboxylate-PEG tetraethylammonium salt as initiator (Scheme 3), as described previously [23]. The theoretical molar mass of the PMLABe block was fixed by the ratio monomer to the initiator and selected at 15,000 g/mol.

In this case also, the polymerization’s reaction was followed by FT-IR, and stopped when the band characteristic of the lactone ring at 1850 cm^−1^ totally disappeared from the FT-IR spectrum. After reaction, the obtained polymers were purified by precipitation in ethanol (elimination of unreacted PEG chains) and characterized by ^1^H NMR (structure and PMLABe molar mass—Figure SI 3.3) and SEC (molar mass and dispersity). Due to the presence of the PEG block with a known molar mass, it is possible to determine, by ^1^H NMR, the molar mass of the PMLABe block [23] (Table 2).

As shown by results gathered in Table 2, the expected amphiphilic block copolymer P3 was successfully obtained.

We have included this PEG_45_-*b*-PMLABe_73_ amphiphilic block copolymer in the present study because it spontaneously forms stable core-shell NPs that are particularly suitable as a drug delivery system [22,23,30,31].

Enzymatic polymerization: The enzymatic polymerization of unsubstituted lactones (γ-butyrolactone, δ-valerolactone, ε-caprolactone, and others) has been widely studied since the 1980s–1990s [48,49]. On the other hand, the enzymatic polymerization of substituted lactones, such as MLABe, is much less described [50]. The first enzymatic polymerization of MLABe was carried out in 1996 by Matsumura et al. [51]. More recently, Panova et al. [52] have also studied the enzymatic polymerization of propyl malolactonate in the presence of Candida rugosa lipase. In this context, we have first replicated Matsumura’s work, and we have next tried to improve it. Thus, the effect of various parameters (enzyme/MLABe ratio, aqueous phase/organic phase ratio, and stirring rate) was studied. However, given the large number of parameters that can influence the reaction, we have chosen to use a design of experiment approach to find the best conditions leading to well-defined high molar mass enzymatic PMLABe [43]. Using such an approach, we were able to find reaction conditions under which the expected enzymatic PMLABe were reproducibly synthesized [43].

**Scheme 4:**
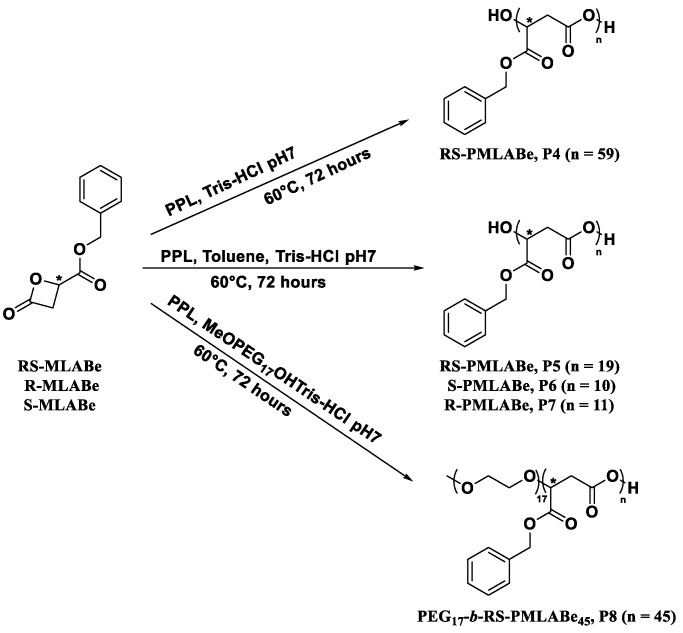
Enzymatic synthesis of PMLABe P4, P5, P6, and P7, and of PEG_17_-b-PMLABe_45_ P8.

Therefore, we used the conditions that we previously defined to synthesize racemic and optically active PMLABe as well as an amphiphilic PEG-*b*-PMLABe block copolymer by the ring-opening polymerization of racemic or optically active MLABe in the presence of PPL as a catalyst (Table 3, Scheme 4). The enzymatic polymerizations were conducted either in the presence or absence of toluene. After 72 h of reaction, all of the polymers were purified by precipitation in diethyl ether, and analyzed by ^1^H NMR (structure, Figures SI 3.4–3.8), SEC (molar mass and dispersity) and polarimetry.

Well-defined enzymatic PMLABe derivatives were obtained as highlighted by the relatively low dispersity values of around 1.5 (Table 3). Moreover, the molar masses of the polymers can be modulated by adding toluene or not during the enzymatic polymerization reaction. Indeed, the molar mass of the PMLABe P4 that was obtained by enzymatic polymerization of RS-MLABe in the absence of toluene is higher than the one of RS-PMLABe P5 obtained by the same reaction conducted in the presence of toluene (Table 3). The S-PMLABe P6 was obtained by enzymatic polymerization of the R-MLABe in toluene, while the R-PMLABe P7 was prepared by the ring-opening polymerization of the S-MLABe in toluene in presence of PPL as the catalyst.

It is important to note that enzymatic ring-opening polymerization leads to an inversion of configuration of the asymmetric carbon. This is similar to the chemical ring-opening polymerization of the optically active MLABe [53], as demonstrated by the value of the specific rotary power for the enzymatic S-PMLABe P6 polymer, which is itself similar to the one measured by the chemical S-PMLABe P2 compound in both the value and sign. Both polymers P6 and P2 were synthesized by the ring-opening polymerization of the R-MLABe in presence of PPL and tetraethylammonium benzoate, respectively. Such an observation suggested that the MLABe ring-opening in the presence of the PPL occurs through an O-alkyl cleavage without the intervention of the serine from the catalytic triad. This result is quite surprising, especially if compared to what is usually observed for unsubstituted lactones. Indeed, the ring-opening polymerization of unsubstituted lactones in the presence of lipases has been described to occur via the canonical mechanism involving the activated serine of the catalytic triad, leading to a ring opening through an O-acyl cleavage [37,38,39]. Our preliminary results rather support the conclusion that the β-substituted β-lactones, as in the case of the AROP reactions, are polymerized by lipases through an O-alkyl cleavage. However, the exact mechanism needs to be further studied, and we undertake several experiments using a serine–knocked-out lipase, which is currently under synthesis, to confirm that the ring-opening polymerization of β-substituted β-lactones by lipases occurs through an O-alkyl cleavage without involvement of the catalytic serine. 

Although the mechanism of polymerization is not clearly established, the enzymatic polymers obtained (P4 to P7) were used to prepare the corresponding NPs. 

The last enzymatic synthesis of an amphiphilic block copolymer was realized in toluene solution in the presence of PPL as the catalyst and an α-methoxy,ω-hydroxyl PEG_17_. The obtained polymer PEG_17_-*b*-PMLABe_45_ (P8) was characterized by ^1^H NMR (structure, Figure SI 3.8) and SEC (molar mass and dispersity). The SEC analysis indicated the formation of a block copolymer as a result of the presence of only one peak with a quite narrow distribution (Đ = 1.50, Table 3). 

### 3.3. Preparation and Characterization of the Nanoparticles

The methodology that was used to prepare the NPs starting from the eight polymers that were previously produced was based on the nanoprecipitation method as previously described [44,45] (see materials and methods section).

For the NPs loaded with the DiR fluorescent probe, the rate of DiR encapsulation was determined. To measure the amount of DiR encapsulated in the nanoparticles, 80 μL of the solution of nanoparticles encapsulating the DiR were added to 320 μL of DMF. The optical density of these solutions was then measured at 670 nm. The amount of encapsulated DiR was determined using a calibration curve of DiR solutions at different concentrations in DMF/water (80/20) (Figure SI 4).

Knowing the initial amount of DiR, we were able to determine the rate of DiR encapsulation that varied between 45–95%, with quantitative encapsulation rates for NPs 2, NPs 3, and NPs 8 (Table 4). 

Table 4 also summarizes the hydrodynamic diameters (Dh) and dispersities (PDIs), measured by DLS, of the PMLABe-based NPs. All of the NPs have hydrodynamic diameters between 83–200 nm, which are values expected for NPs based on either hydrophobic or amphiphilic (co)polymers encapsulating a fluorescent probe such as the DiR. The samples are relatively monodisperse, since the PDIs values were all between 0.13–0.26.

In this case, the synthesized PMLABe polymers were dissolved in 1,4-dioxane. This solution was quickly added in distilled water to trigger nanoprecipitation and auto-assembly of the polymers into NPs. The final concentration of polymers was 2.5 mg/mL. Table 5 summarizes the hydrodynamic diameters (Dh) and PDIs of the corresponding NPs used for the cytotoxicity study.

The hydrodynamic diameters (Dh) of NPs were found in the range of 50 nm to 120 nm (average diameter of 102 nm ± 19 nm). The smallest Dh was measured for NPs 3 produced with the amphiphilic block copolymer PEG_45_-*b*-PMLABe_73_ P3, harboring the longest PEG chain. Such a result is in good agreement with the already observed characteristics of NPs based on PEGylated amphiphilic block copolymers [23]. All of the PDIs’ values are lower than 0.3, indicating a good size distribution with the exception of the NPs 2 formulation, for which the PDI is slightly greater than 0.3. Nevertheless, we considered that all of the prepared formulations meet the conditions for the in vitro cytotoxicity assays.

### 3.4. In Vitro Assays

#### 3.4.1. In Vitro Cytotoxicity Assays

In order to evaluate the biocompatibility of the PMLABe-based NPs, we incubated human hepatoma HepaRG cells with the 8 NPs in a wide range of concentrations. HepaRG cells are bipotent hepatic progenitors that can be differentiated into hepatocyte-like cells [54] expressing most of the major hepatic detoxifying enzymes [55]. These metabolically competent hepatocyte-like cells are used worldwide as an in vitro model for studying the metabolism [56] and toxicity of xenobiotics [57,58,59], including exposure to NPs [31,47,60].

**Figure 1:**
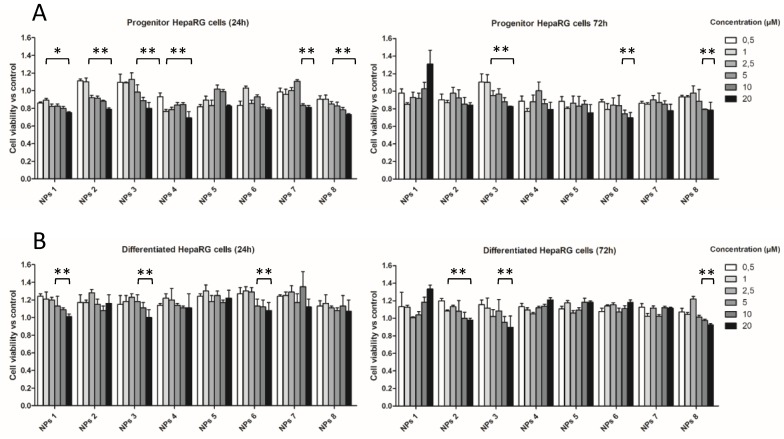
MTT assays (viability assay) in progenitor (**A**) and hepatocyte-like HepaRG cells (differentiated cells, **B**) incubated with PMLABe-based nanoparticles at 24 h (24 h) and 72 h (72 h). Statistical analyses: * *p* < 0.05; ** *p* < 0.01 versus non treated cells, three independent experiments.

Proliferating progenitor and quiescent differentiated HepaRG cells were incubated with NPs for 24 h and 72 h at concentrations ranging from 0.5 µM to 20 µM, and the cell viability was assayed using the MTT assay (Figure 1). The exposure of progenitor cells during 24 h triggered a moderate and partially dose-dependent decrease in MTT activity for NPs 1, 2, 3, 4, 7, and 8 compared to that measured in control cultures without nanoparticles (Figure 1A). This effect was less significant at 72 h. For these NPs, the highest concentrations in polymers (10 µM and 20 µM) reduced the cell viability by less than 30%, and the half-maximal inhibitory concentration (IC_50_) was not reached for these amounts of NPs. The exposure of hepatocyte-like HepaRG cells that exhibit a higher ability to metabolize xenobiotics, showed a weaker decrease in MTT activities (Figure 1B). 

Together, these data demonstrated the mild toxicity of these PMLABe-based NPs following incubation with HepaRG cells, which is in agreement with our previous reports describing the moderate effects on total cell numbers following the exposure of various human cell lines, including the Huh7 hepatoma cell line with NPs 1 and NPs 3 [23]. Interestingly, in this work, we compared the effects of NPs prepared from polymers synthesized by chemical (NPs 1 to 3) and enzymatic (NPs 4 to 8) polymerization. Our data indicate that mild effects on cell viability were observed with both types of NPs based on either enzymatic or chemically synthesized PMLABe. Morever, the use of NPs constituted by PMLABe derivatives prepared through enzymatic synthesis did not really improve the cell viability in comparison to what is observed with NPs based on chemical PMLABe dervatives. 

#### 3.4.2. In Vitro Cells Uptake Assays

**Figure 2:**
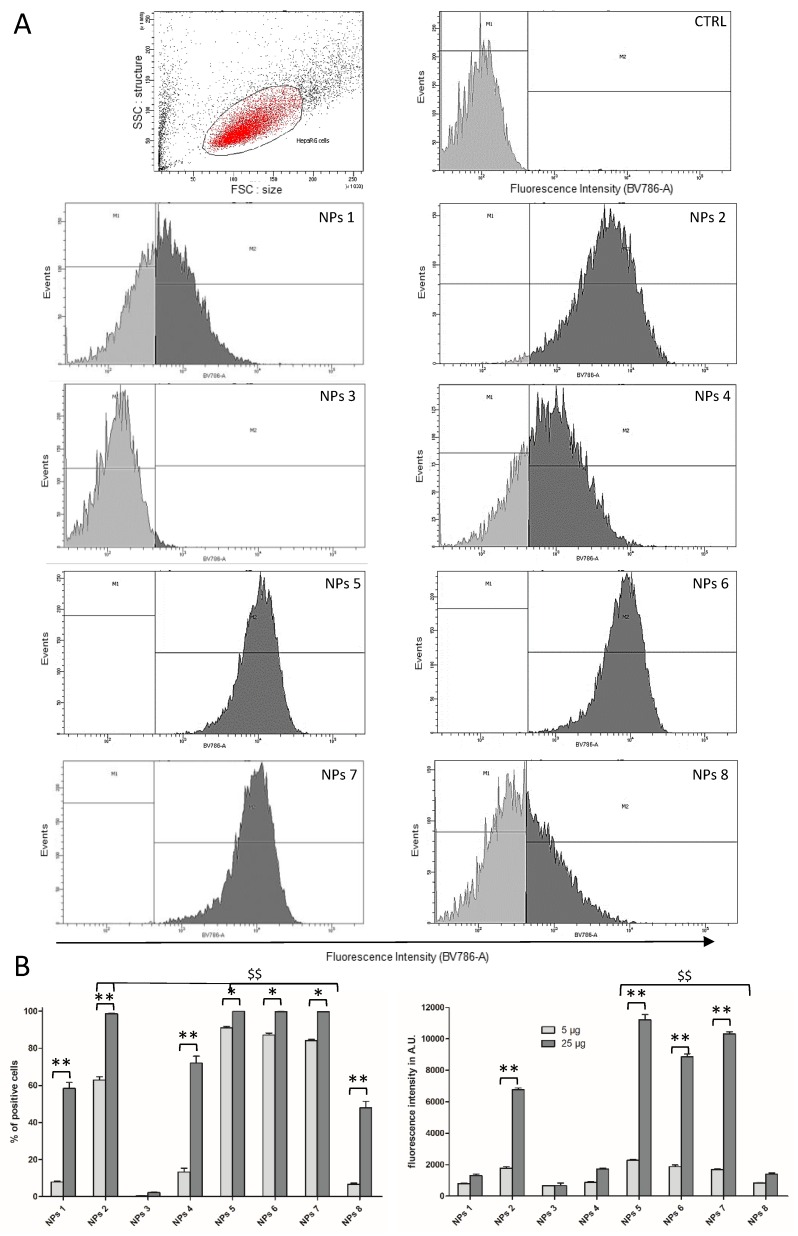
(**A**) Dot plot (control HepaRG cells) and histograms used to detect negative (M1 gate) and positive (M2 gate) cells and determine the mean of fluorescence in control (CTRL) and cells incubated with nanoparticles (NPs) 1 to 8; (**B**) Quantification of percentages of positive cells and mean of fluorescence in progenitor HepaRG cells incubated with PMLABe-based nanoparticles for 24 h. Statistical analyses: ** *p* < 0.01: cells incubated with 5 µg/mL of polymers versus cells incubated with 25 µg/mL; $$ < 0.01: highest percentages of positive cells or mean of fluorescence versus all the other conditions.

**Figure 3:**
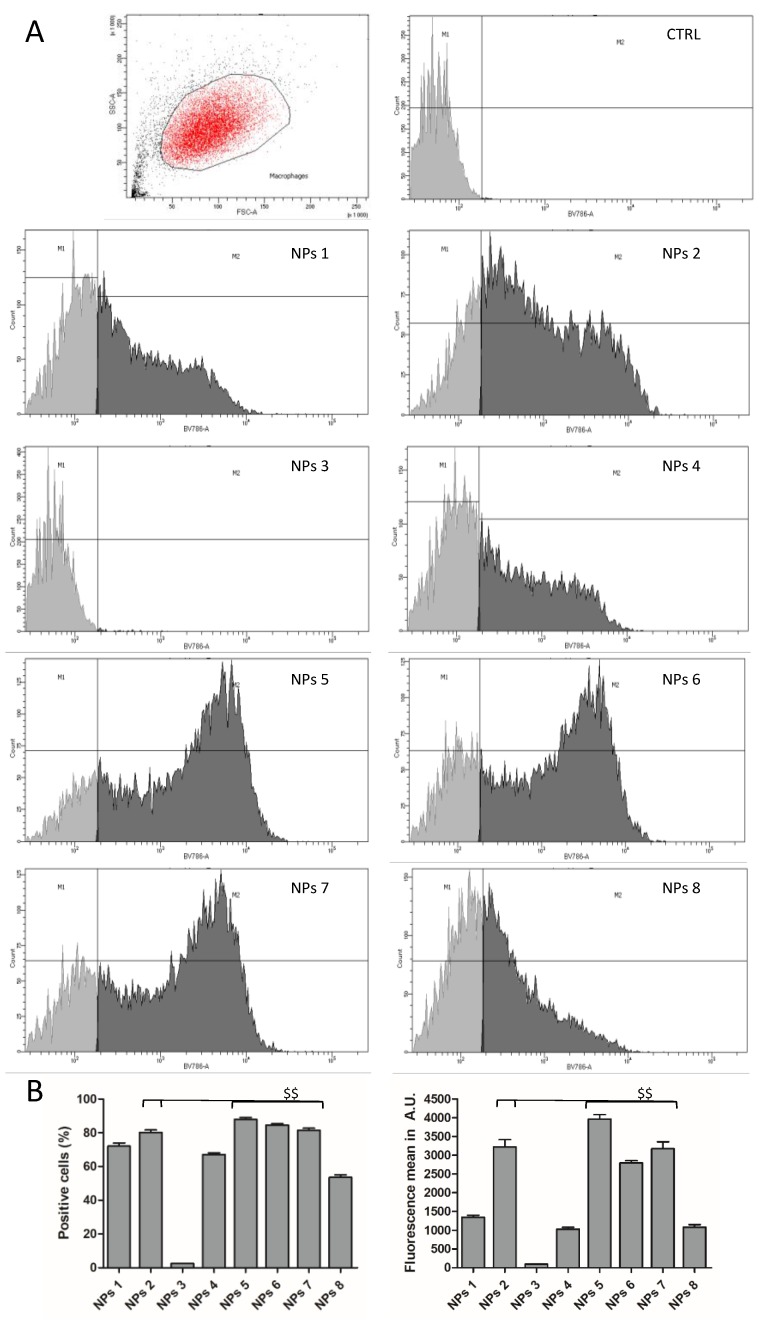
(**A**) Dot plot (control macrophages) and histograms used to detect negative (M1 gate) and positive (M2 gate) cells and determine the mean of fluorescence in control (CTRL) and cells incubated with NPs 1 to 8; (**B**) Quantification of percentages of positive cells and mean of fluorescence in macrophages incubated with PMLABe-based nanoparticles for 24 h. Statistical analyses: $$ < 0.01: highest percentages of positive cells or mean of fluorescence versus all the other conditions.

The NPs’ cell uptake by HepaRG cells (Figure 2) and human macrophages (Figure 3) of PMLABe-based nanoparticles was monitored by the detection of the cells that internalized NPs encapsulating the DiR lipophilic fluorescent dye using flow cytometry.

The flow cytometry analysis (Figure 2A and Figure 3A) was performed on single cells gated on dot plots with size of events (*x* axis, forward scatter, FSC-A) and structure (*y* axis, side scatter, SSC-A). HepaRG cells and macrophages non-incubated with NPs were used to determine the endogenous cell fluorescence and the M1 gate corresponding to negative cells. The M2 gate defined the positive cell populations that internalized fluorescent NPs. 

We first compared the HepaRG cell uptake of NPs using 5 µg/mL and 25 µg/mL of polymers (Figure 2B). At 5 µg/mL, the cell uptake was very low for NPs 1, 3, 4, and 8, with less than 10% of positive cells, while NPs 2, 5, 6, and 7 were internalized in more than 60% of the HepaRG cells. At 25 µg/mL, the percentages of positive cells and the intensity of fluorescence, reflecting the intracellular accumulation of NPs, were considerably enhanced with high levels of internalization for NPs 1, 2, 4, 5, 6, and 7, indicating that this concentration of polymers and the corresponding amounts of NPs were more appropriate to visualize the cell uptake. At this concentration, in both cultures of HepaRG cells and macrophages, cellular uptakes of NPs 2, 5, 6, and 7 prepared from PMLABe were very efficient, with at least 80% of positive cells and the highest mean of fluorescence. The efficient internalization of these NPs was not correlated to the molar masses of the corresponding homopolymers, but with small hydrodynamic diameters (Table 4). Interestingly, the cell uptake of NPs 2 was much higher than that of NPs 1, while they exhibit similar molar masses and hydrodynamic diameters. The main difference between these two NPs was that NPs 1 were prepared with RS-PMLABe, while NPs 2 were formulated with S-PMLABe, suggesting that NPs derived from this optically active polymer exhibit a physicochemical feature favoring cell uptake. Furthermore, the highest cell uptake was observed for NPs 5 to 7, which were prepared from homopolymers synthesized by enzymatic polymerization. This data led us to postulate that PMLABe homopolymers obtained by enzymatic polymerization, and with low molar masses (<4000 g/L), formed NPs that are more efficiently internalized than NPs produced from PMLABe polymers synthesized by chemical polymerization. 

In contrast, the percentages of positive cells were much lower for PEGylated NPs 8 (PEG_17_-*b*-PMLABe_45_) with 45% to 55% of positive cells and almost abolished for PEGylated NPs 3 (PEG_45_-*b*-PMLABe_73_) with less than 5% of positive cells. These data are in agreement with the well-established role of the neutral PEG moiety that reduces the opsonization of PEGylated NPs by plasma proteins and their cell internalization.

## 4. Conclusions

In the present work, we have successfully synthesized and characterized a series of eight PMLABe-based polymers through chemical and enzymatic polymerization, formulated the corresponding NPs, studied their cytotoxicity human hepatoma HepaRG cells, and measured their uptake by both primary macrophages and HepaRG cells.

In vitro cell viability assessed by MTT on HepaRG cells evidenced a mild toxicity of the NPs formed from PMLABe polymers at high concentrations/densities of NPs in the culture media of cells. This cytotoxicity is in agreement with our previous reports, and does not evidence a higher biocompatibility of the NPs prepared from polymers synthesized by enzymatic polymerization. These data further demonstrate that the chemical polymerization and the procedure of nanoprecipitation led to the production of biocompatible PMLABe-based NPs. The efficient uptake combined with low cytotoxicity toward HepaRG cells indicates that PMLABe-based NPs are suitable nanovectors for drug delivery that will further be evaluated in vivo to target either hepatocytes or resident liver macrophages.

## Figures and Tables

**Table 1:** Characteristics of the RS-PMLABe (P1) and S-PMLABe (P2).

Polymers	*M*_theoritical_ g/mol	$\bar{M}w$^a^ g/mol	Đ ^a^	[α]_D_^b^
RS-PMLABe, P1	30,000	14,850	1.60	0
S-PMLABe, P2	30,000	n.d.	n.d.	−10.7

^a^ Measured by SEC in THF, standards polystyrene, 40 °C, 1 mL/min; ^b^ Measured by polarimetry in chloroform. n.d.: not determined.

**Table 2:** Characteristics of the PEG_45_-b-PMLABe_73_ (P3).

	*M*_PEG_^a^ g/mol	*M*_PMLABe_Theo g/mol	*M*_PMLABe_ NMR g/mol ^b^	$\bar{M}w$^c^ g/mol	Đ ^c^
PEG_45_-*b*-PMLABe_73_, P3	2000	15,000	15,040	9600	1.30

^a^ Molar mass given by the supplier PEGIris Biotech. ^b^ Determined by ^1^H NMR. ^c^ Measured by size exclusion chromatography (SEC) in THF, standards polystyrene, 40 °C, 1 mL/min.

**Table 3:** Characteristics of RS-PMLABe (P4 and P5), S-PMLABe (P6), R-PMLABe (P7), and PEG_17_-b-PMLABe_45_ (P8) obtained by enzymatic polymerization in presence of porcine pancreatic lipase (PPL) as the catalyst.

Polymer	Toluene	$\bar{M}w$^a^ g/mol	Đ ^a^	[a]_D_^c^
RS-PMLABe, P4	No	12,250	1.40	0
RS-PMLABe, P5	Yes	3850	1.50	−3
S-PMLABe, P6	Yes	2000	1.50	−11
R-PMLABe, P7	Yes	2300	1.40	+18
PEG_17_-*b*-PMLABe_45_, P8	Yes	9650	1.50	-

^a^ Measured by size exclusion chromatography (SEC) in THF, standards polystyrene, 40 °C, 1 mL/min; ^b^ Determined by ^1^H NMR; ^c^ Measured by polarimetry in chloroform.

**Table 4:** Characteristics of the prepared NPs loaded with DiR formulated for the cell uptake studies.

Entry	Polymers	Way of Synthesis	Dh nm	PDI	DiR Encapsulation Rate %
NPs 1	RS-PMLABe, P1	Chemical	195	0.19	61
NPs 2	S-PMLABe, P2	Chemical	170	0.26	Quantitative
NPs 3	PEG_45_-*b*-PMLABe_73_, P3	Chemical	83	0.22	Quantitative
NPs 4	RS-PMLABe, P4	Enzymatic	200	0.13	45
NPs 5	RS-PMLABe, P5	Enzymatic	125	0.19	83
NPs 6	S-PMLABe, P6	Enzymatic	145	0.19	95
NPs 7	R-PMLABe, P7	Enzymatic	130	0.16	94
NPs 8	PEG_17_-*b*-PMLABe_45_, P8	Enzymatic	185	0.24	Quantitative

Preparation of nanoparticles for the in vitro cytotoxicity assays. PDI: polydispersity index.

**Table 5:** Characteristics of the nanoparticles prepared for the in vitro cytotoxicity assays.

Entry	Polymers	Way of Synthesis	Dh nm	PDI
NPs 1	RS-PMLABe, P1	Chemical	72	0.18
NPs 2	S-PMLABe, P2	Chemical	122	0.32
NPs 3	PEG_45_-*b*-PMLABe_73_, P3	Chemical	51	0.16
NPs 4	RS-PMLABe, P4	Enzymatic	113	0.28
NPs 5	RS-PMLABe, P5	Enzymatic	96	0.17
NPs 6	S-PMLABe, P6	Enzymatic	121	0.19
NPs 7	R-PMLABe, P7	Enzymatic	107	0.18
NPs 8	PEG_17_-*b*-PMLABe_45_, P8	Enzymatic	83	0.17

## Supplementary Materials

The supplementary materials are available online at http://www.mdpi.com/2073-4360/10/11/1244/s1.
