# Supplementary material for: Cell Uptake and Biocompatibility of Nanoparticles Prepared from Poly(benzyl malate) (Co)polymers Obtained through Chemical and Enzymatic Polymerization in Human HepaRG Cells and Primary Macrophages
Source: Polymers (Basel). 2018 Nov 10;10(11):1244. doi: 10.3390/polym10111244 (PMC6401887; doi:10.3390/polym10111244)

Cell uptake and biocompatibility of nanoparticles prepared from poly(benzyl malate) (co)polymers obtained through chemical and enzymatic polymerization in human HepaRG cells and primary macrophages

Hubert Casajus[1,*], Saad Saba[2,*], Manuel Vlach[2], Elise Vène[2], Catherine Ribault[2], Sylvain Tranchimand[1], Caroline Nugier-Chauvin[1], Eric Dubreucq[3], Pascal Loyer[2,§], Sandrine Cammas-Marion[1,2,§], Nicolas Lepareur[2,4,§.]

[1] Univ Rennes, Ecole Nationale Supérieure de Chimie de Rennes, CNRS, ISCR, UMR 6226, F-35000 Rennes, France.

[2] Univ Rennes, INSERM, INRA, Institut NUMECAN (Nutrition Metabolisms and Cancer) UMR_A 1341, UMR_S 1241, F-35000 Rennes, France.

[3] Montpellier SupAgro, Département des Sciences pour les Agro-Bioprocédés, UMR IATE (INRA - CIRAD - Montpellier SupAgro - Université Montpellier II), Axe Biotechnologie microbienne et enzymatique des lipides et des Agropolymères, 2 place Viala - 34060 Montpellier cedex 2 – France.

[4] Comprehensive Cancer Center Eugène Marquis, F-35000 Rennes, France.

***** These authors contributed equally to the work.

§ Correspondence: pascal.loyer@univ-rennes1.fr (+33-223-233-873), sandrine.marion.1@ensc-rennes.fr (+33-223-238-109), n.lepareur@rennes.unicancer.fr (+33-299-253-144)

Figure SI 1.1. FT-IR spectrum of RS-MLABe

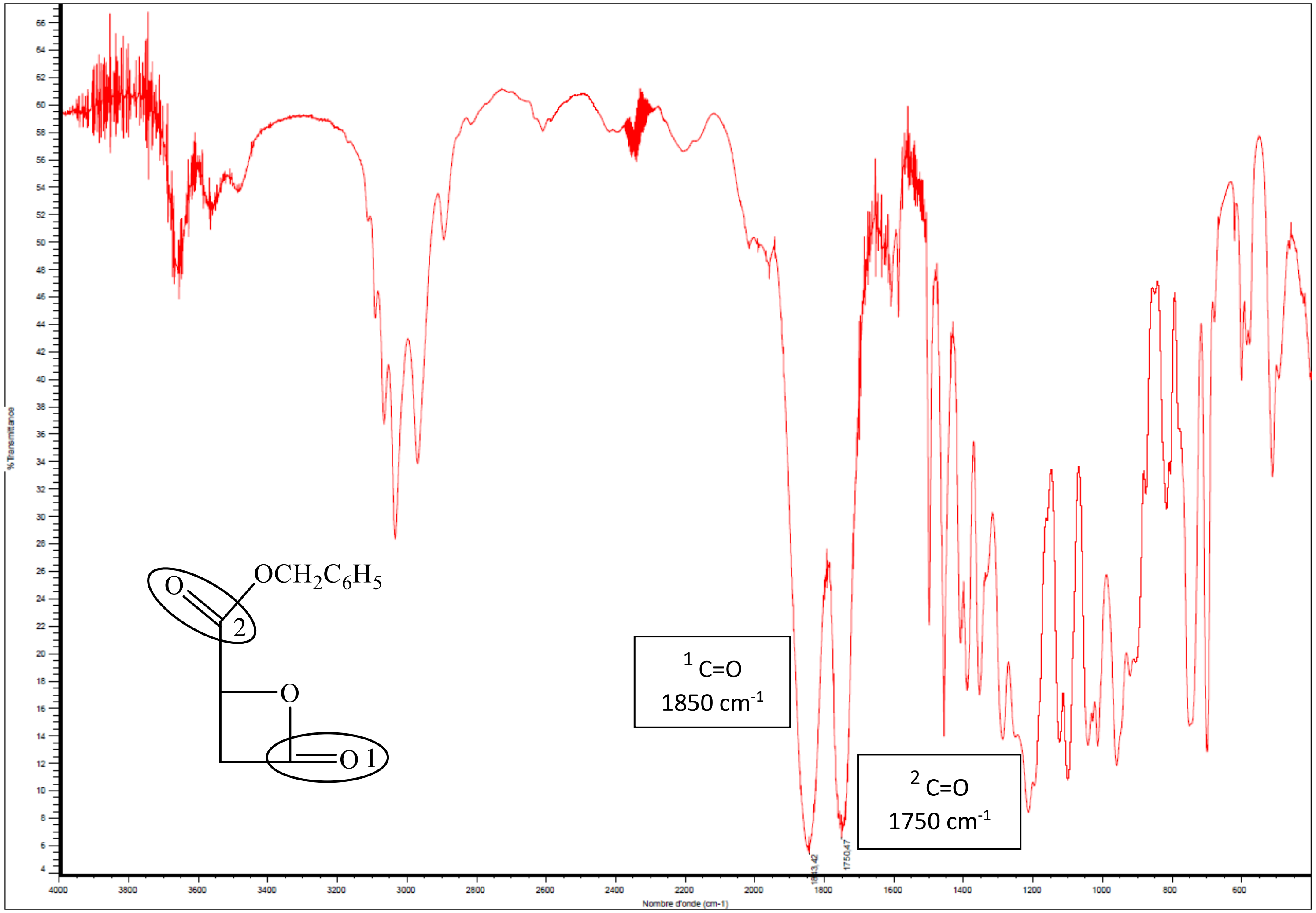

Figure SI 1.2. FT-IR spectrum of S-MLABe

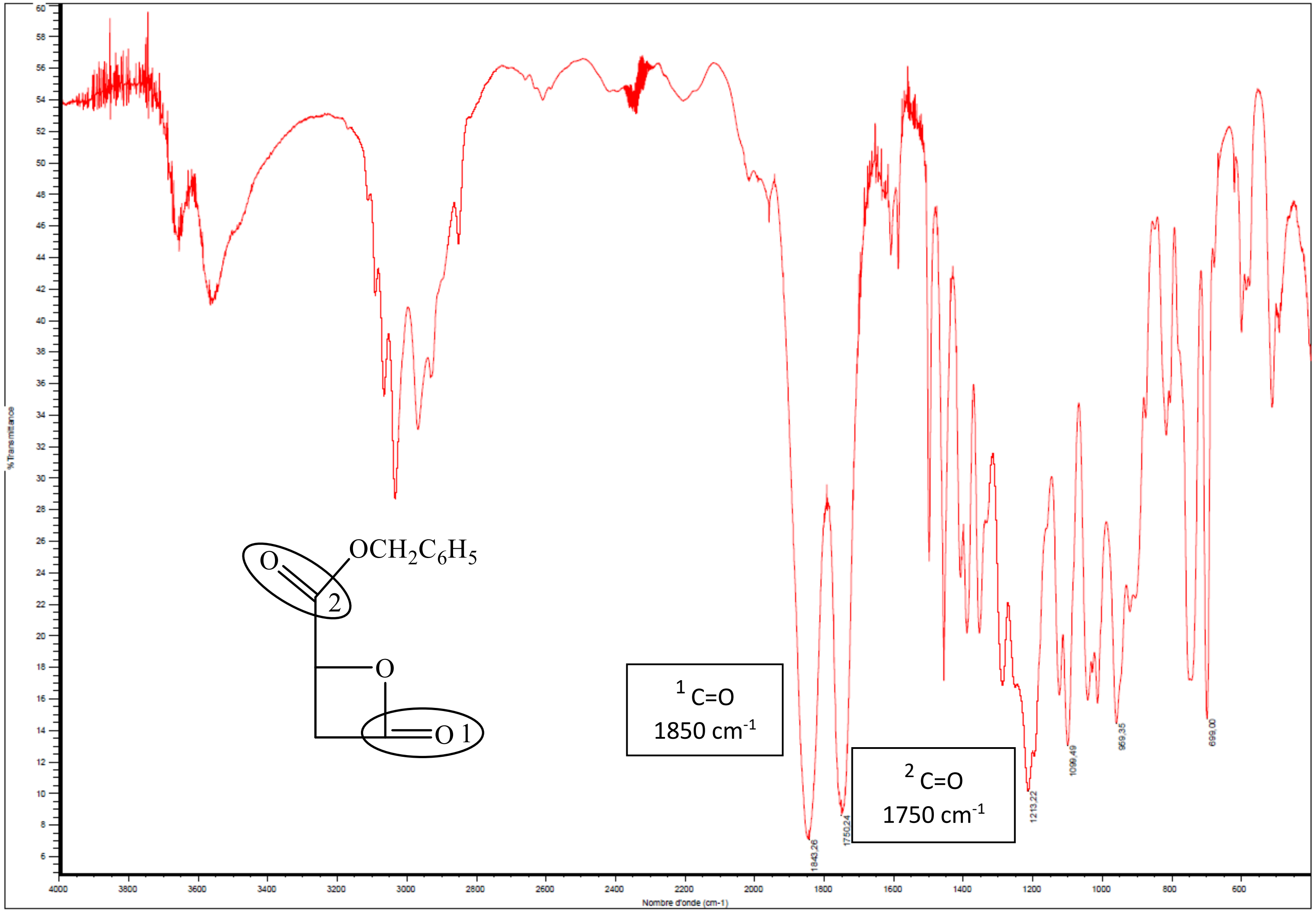

Figure SI 1.3. FT-IR spectrum of R-MLABe

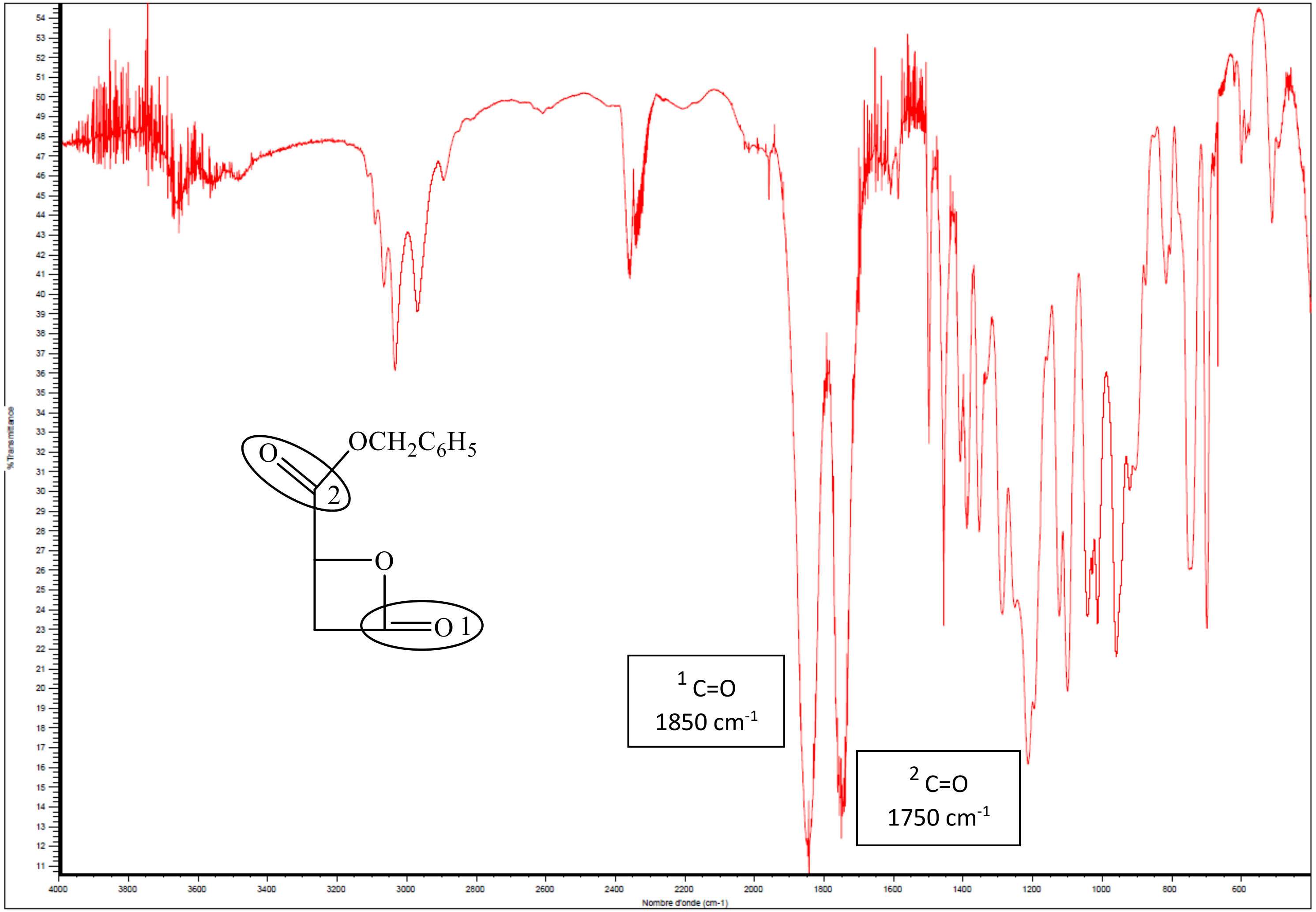

Figure SI 2.1. ^{1}H NMR spectrum of RS-MLABe

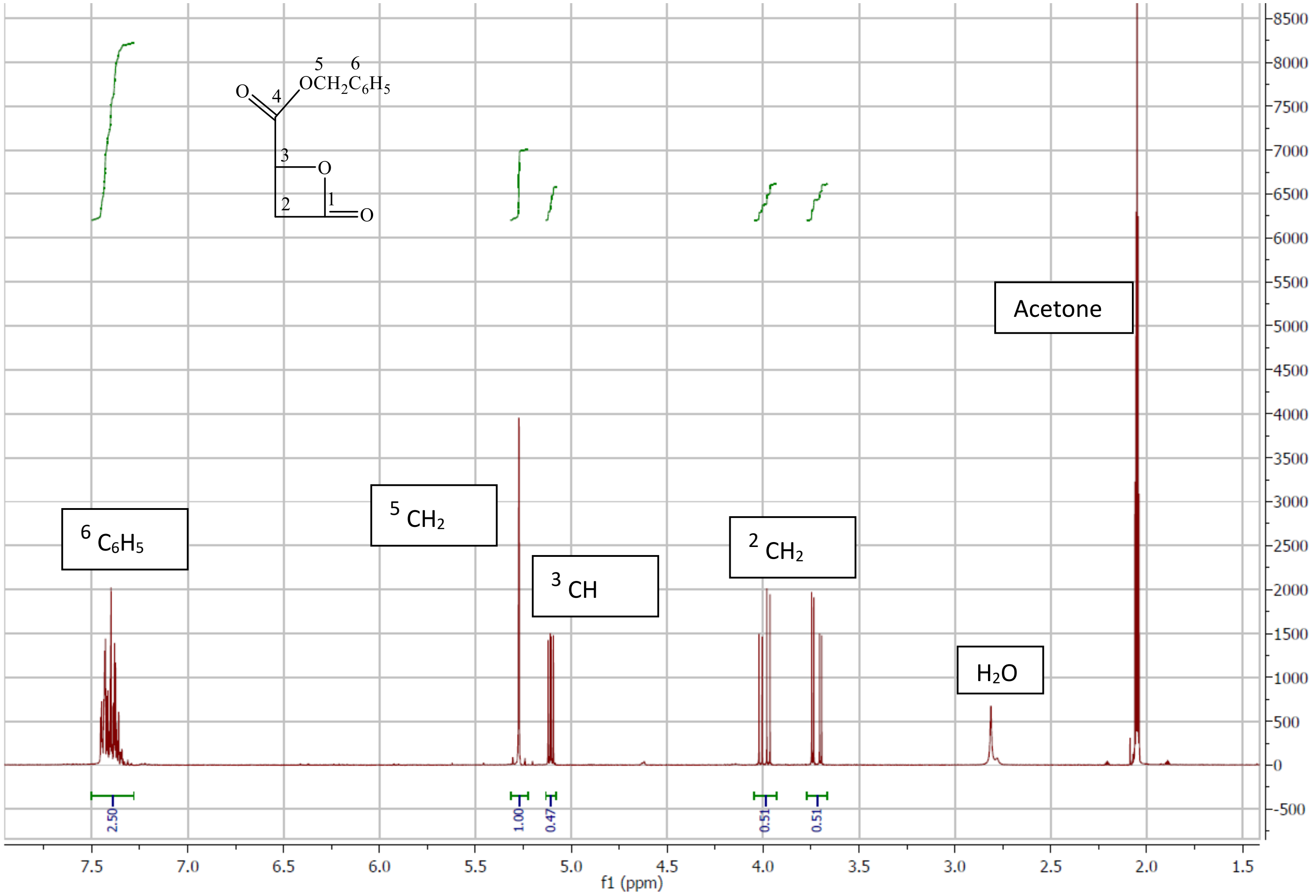

Figure SI 2.2. ^1H NMR spectrum of S-MLABe

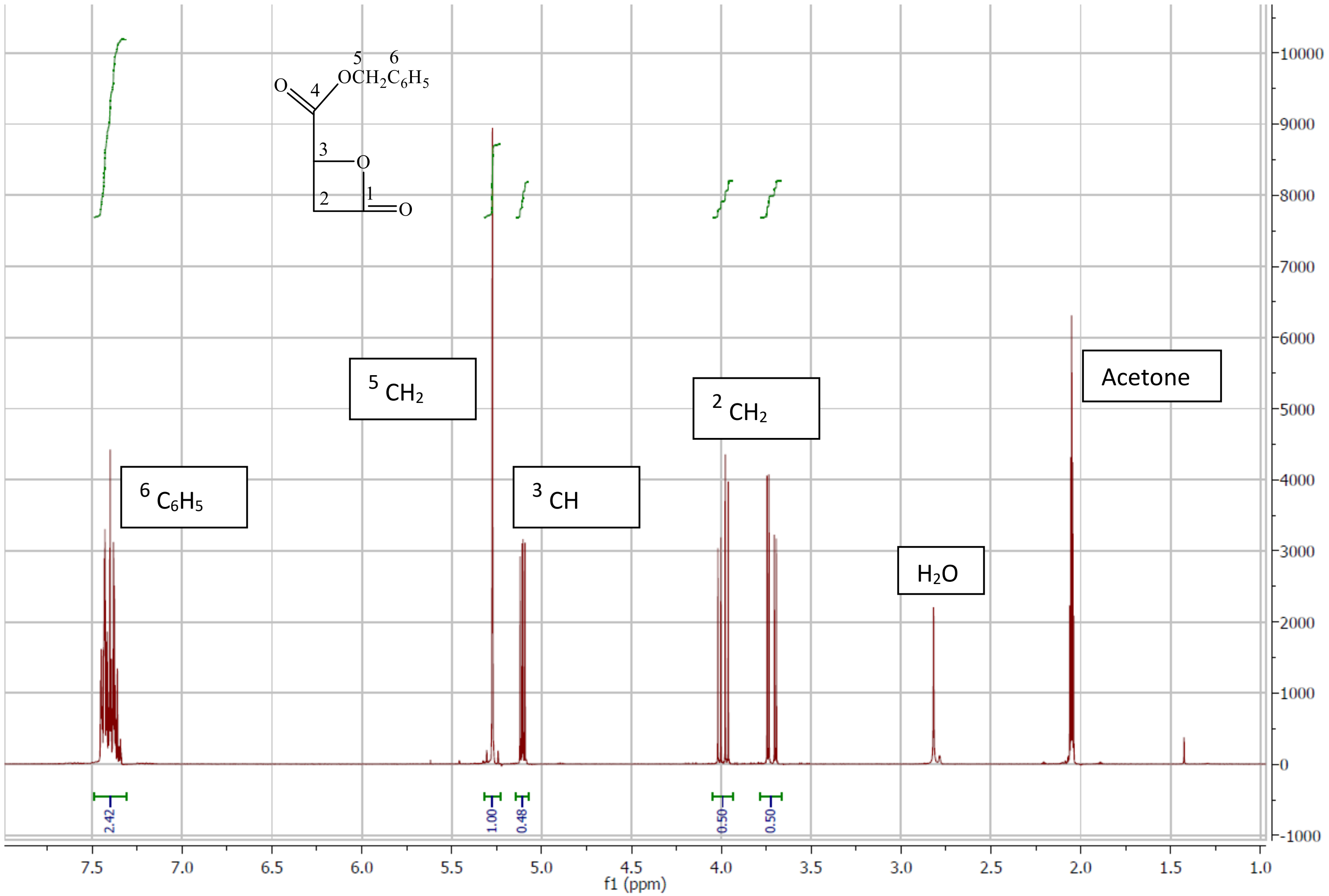

Figure SI 2.3. ^1H NMR spectrum of R-MLABe

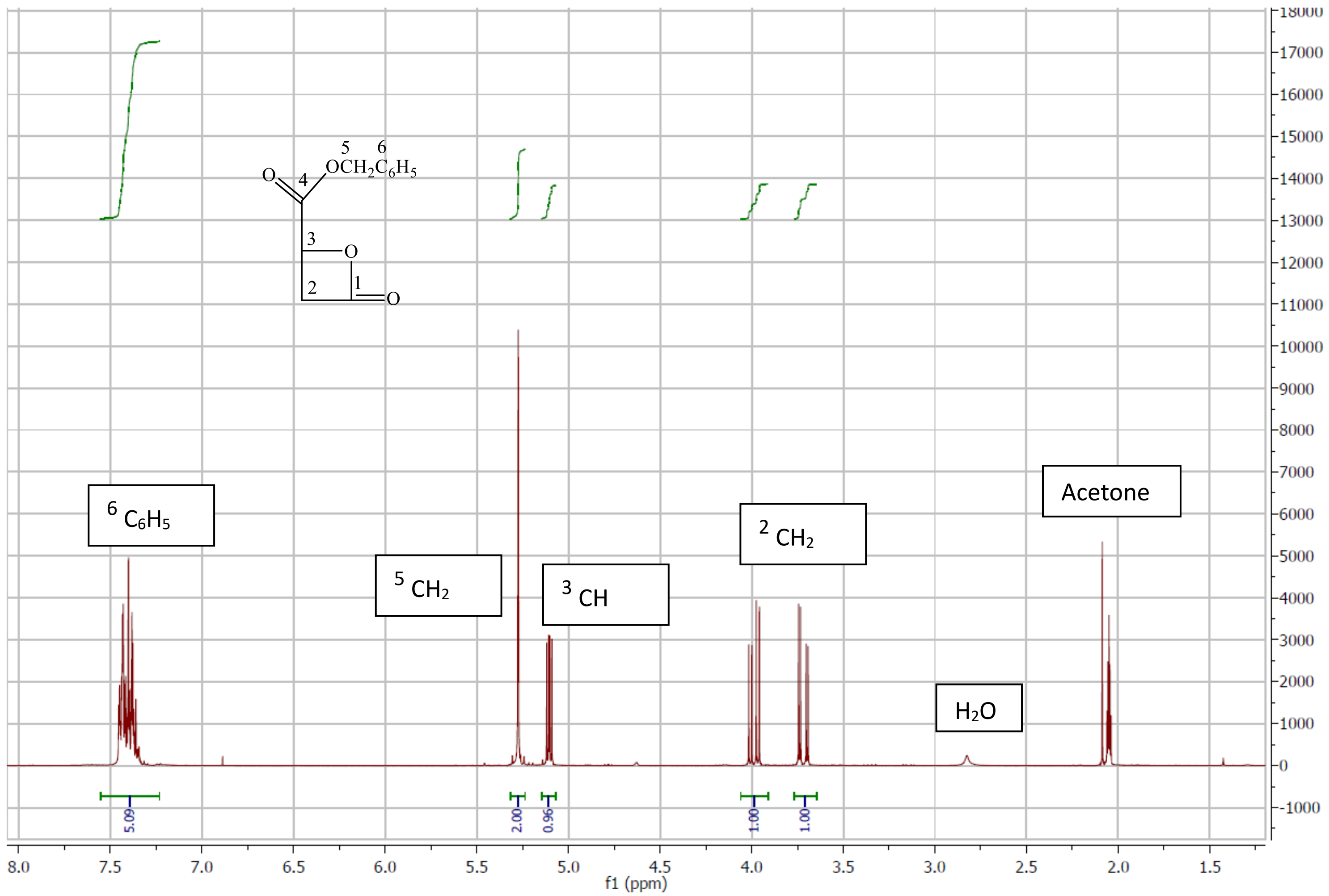

Figure SI 3.1. ^{1}H NMR spectrum of RS-PMLABe P1

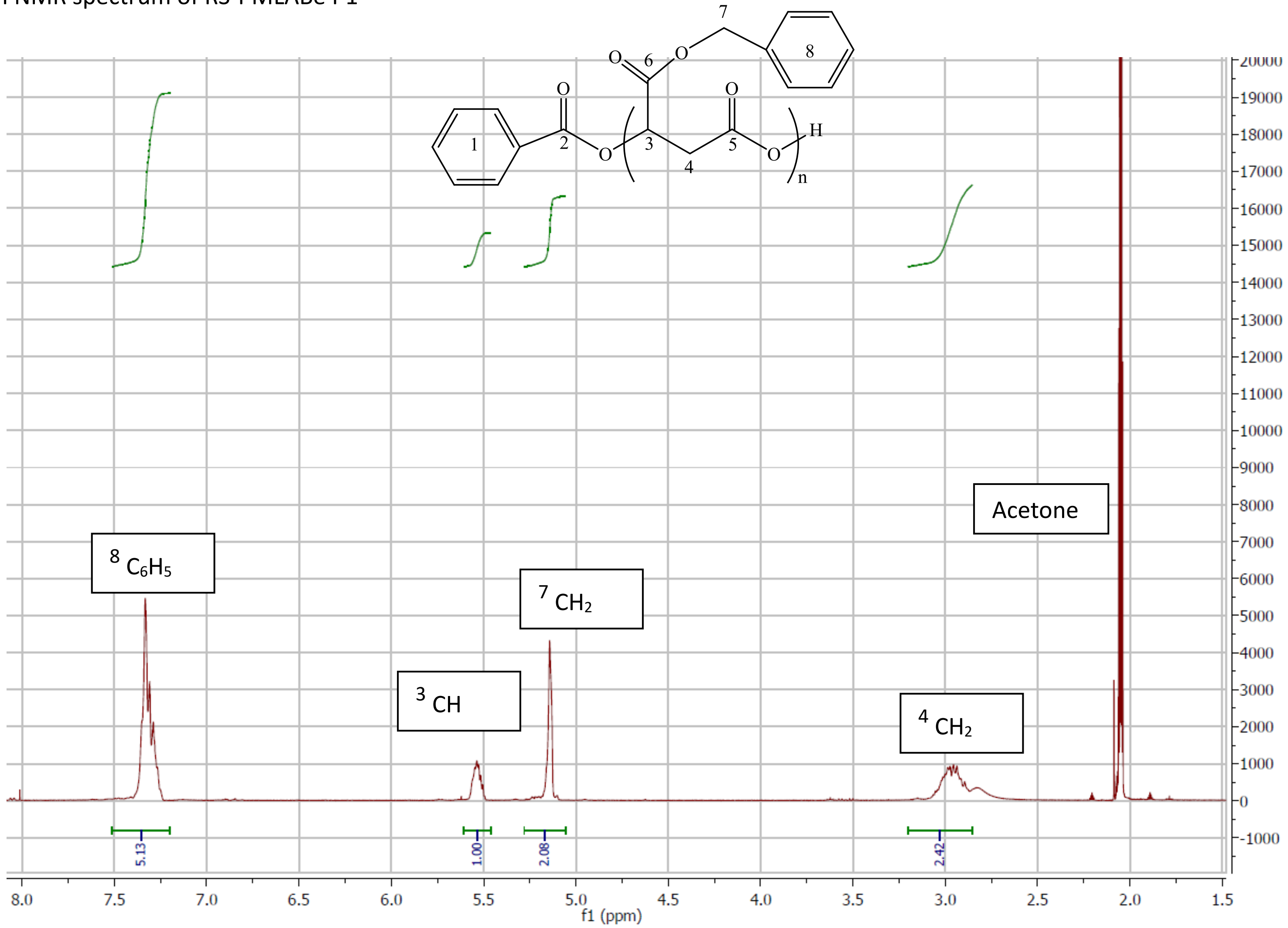

Figure SI 3.2. ^1H NMR spectrum of S-PMLABe P2

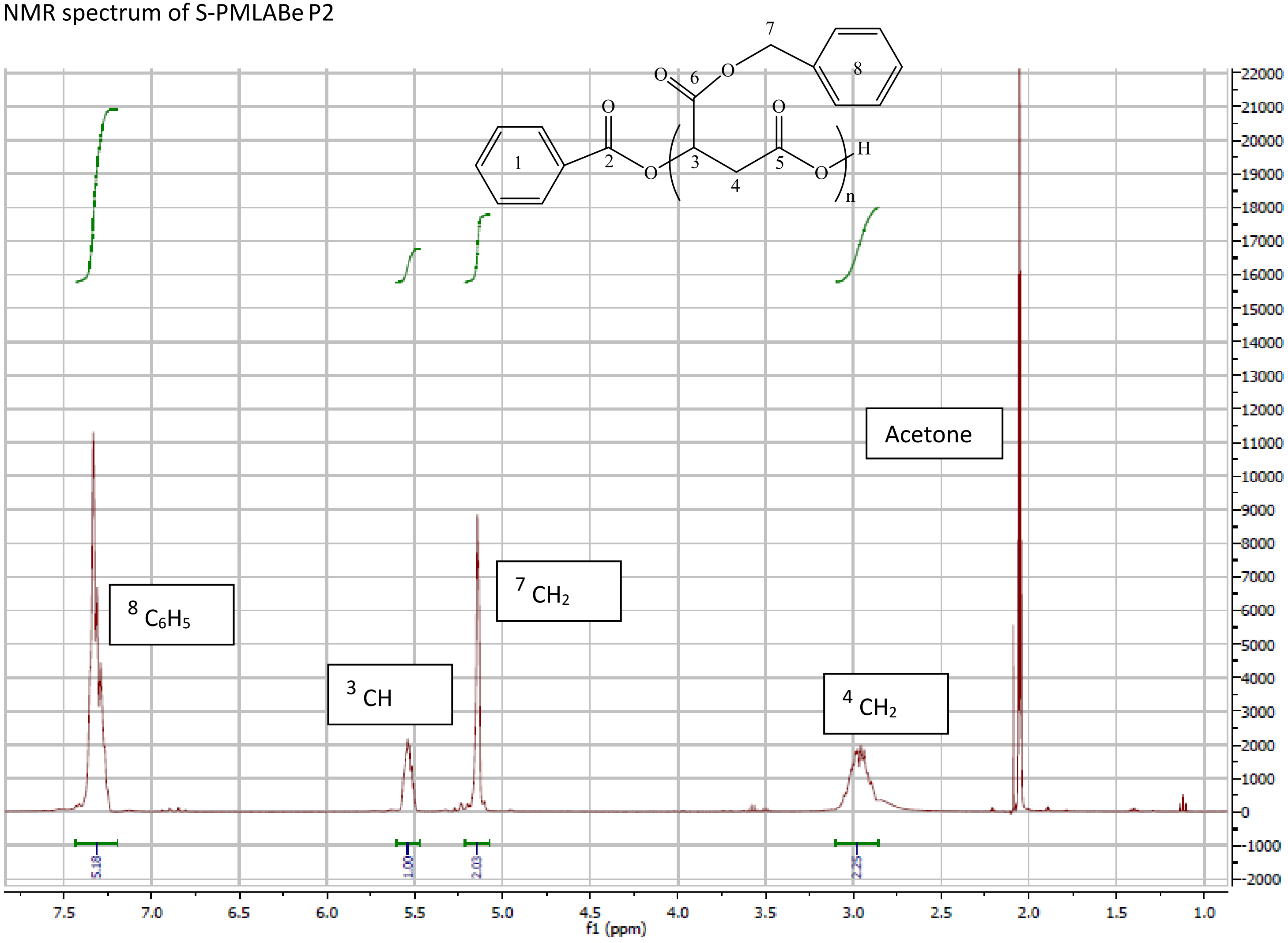

Figure SI 3.3. ^{1}H NMR spectrum of chemical PEG$_{45}$-*b*-RS-PMLABe$_{73}$ P3

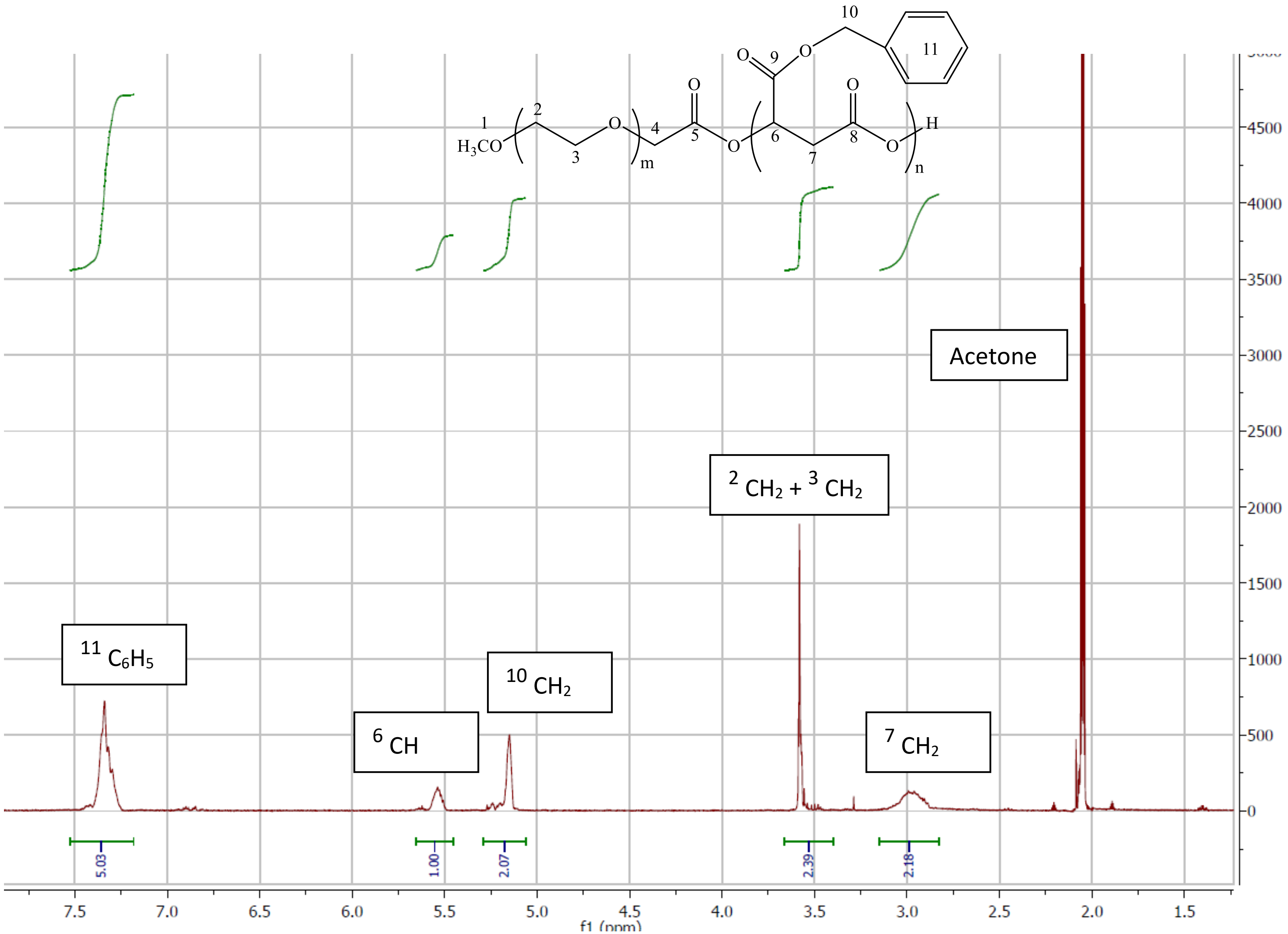

Figure SI 3.4. ^{1}H NMR spectrum of enzymatic RS-PMLABe P4

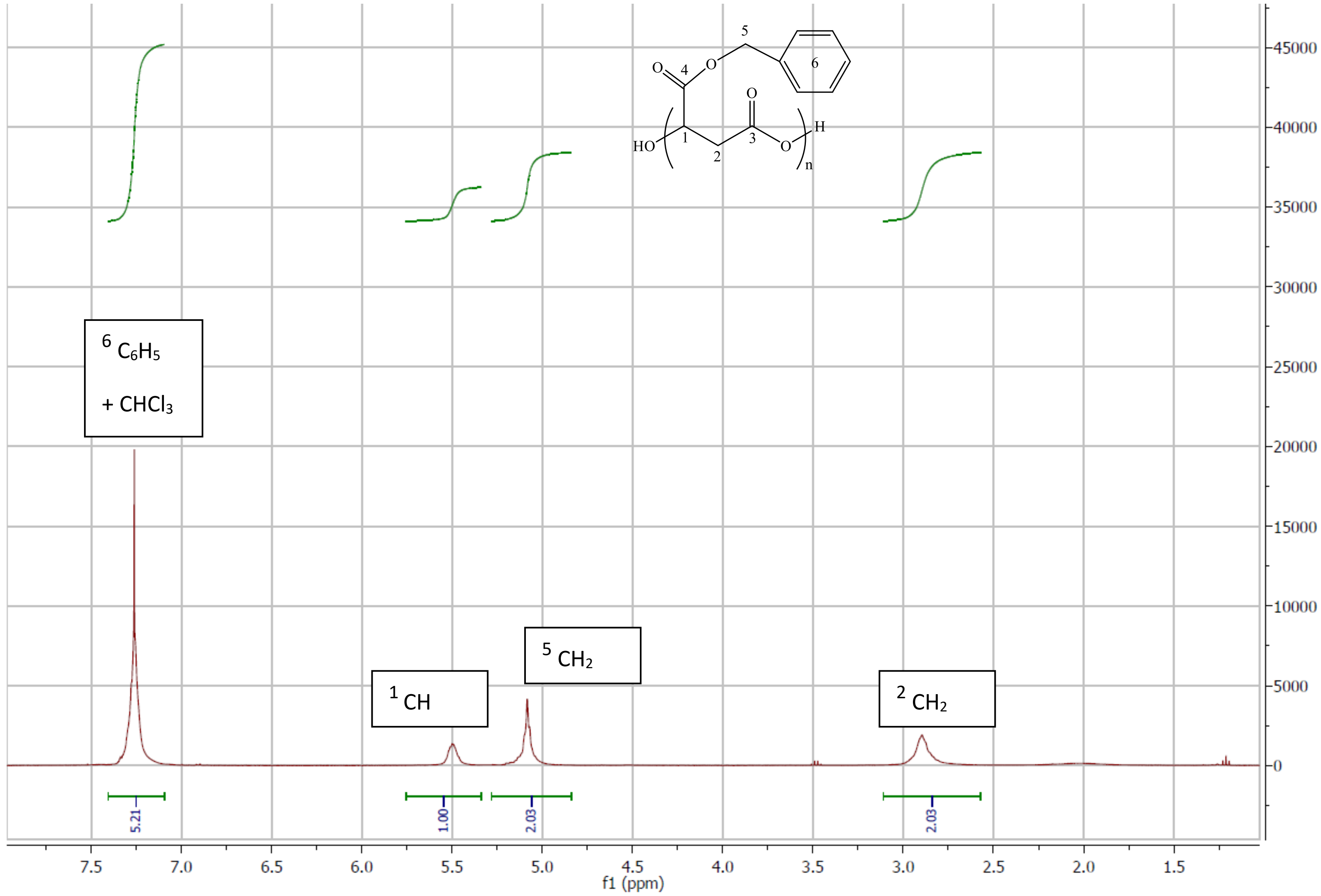

Figure SI 3.5. ^{1}H NMR spectrum of enzymatic RS-PMLABe P5

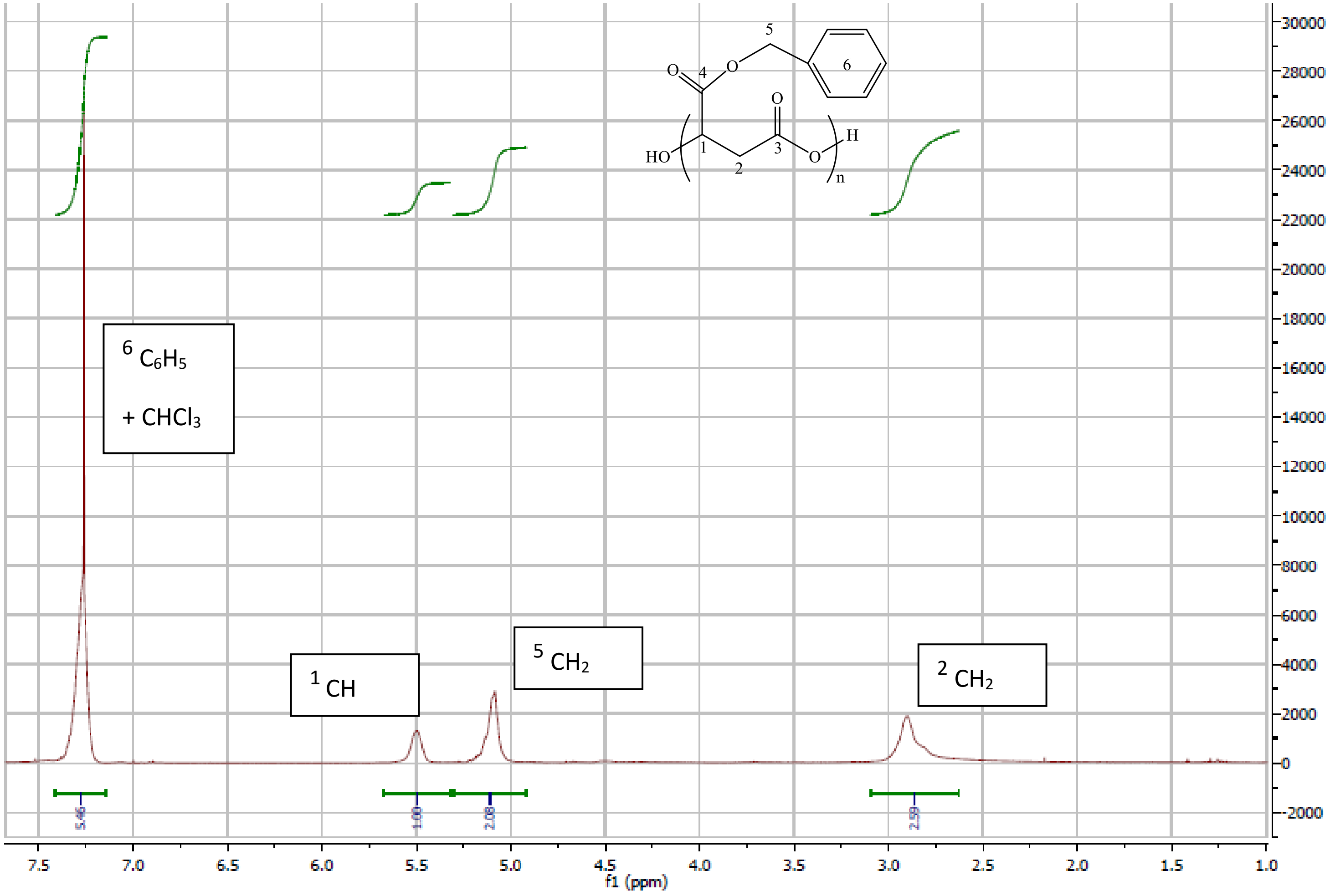

Figure SI 3.6. ^{1}H NMR spectrum of enzymatic S-PMLABe P6

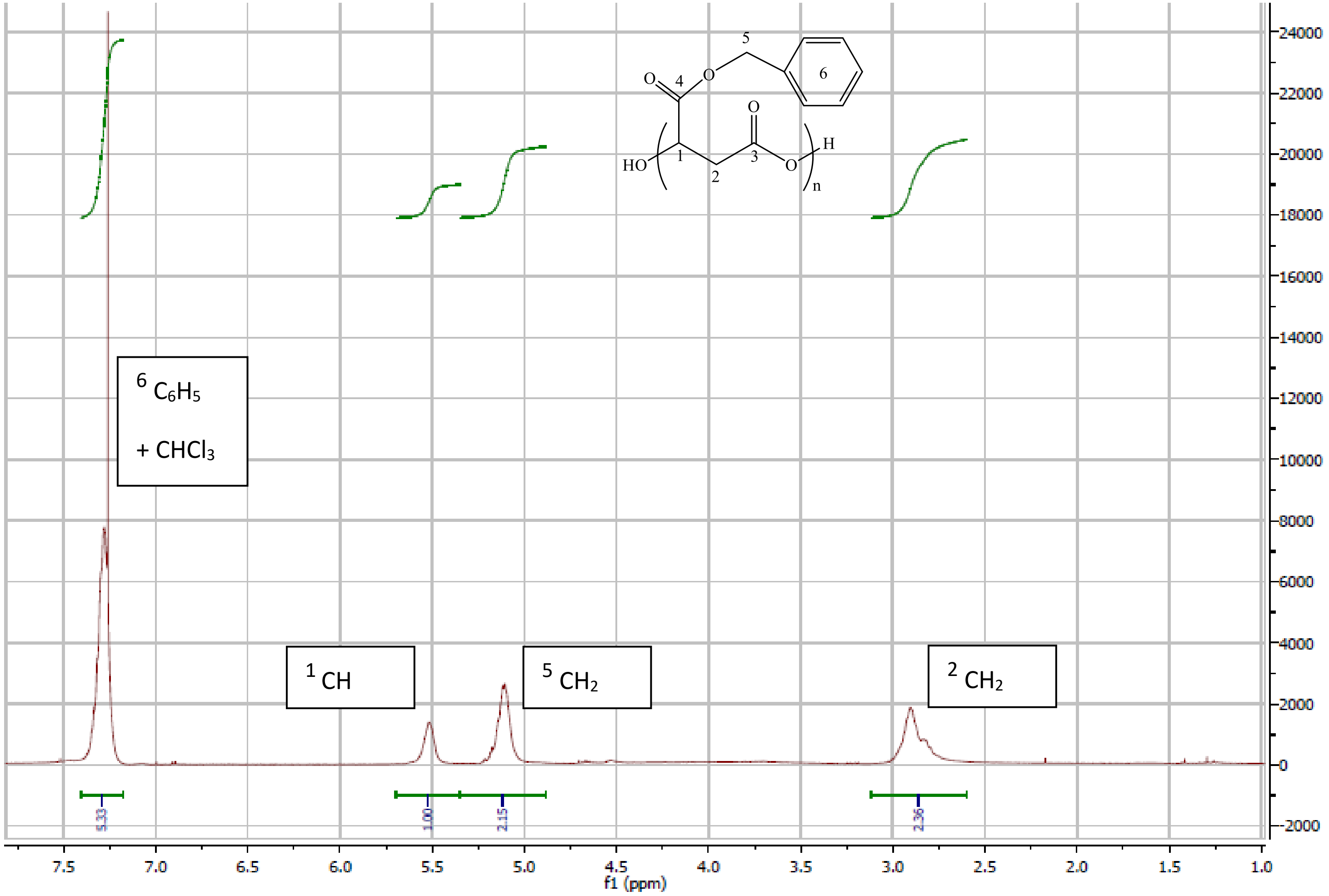

Figure SI 3.7. ^{1}H NMR spectrum of enzymatic R-PMLABe P7

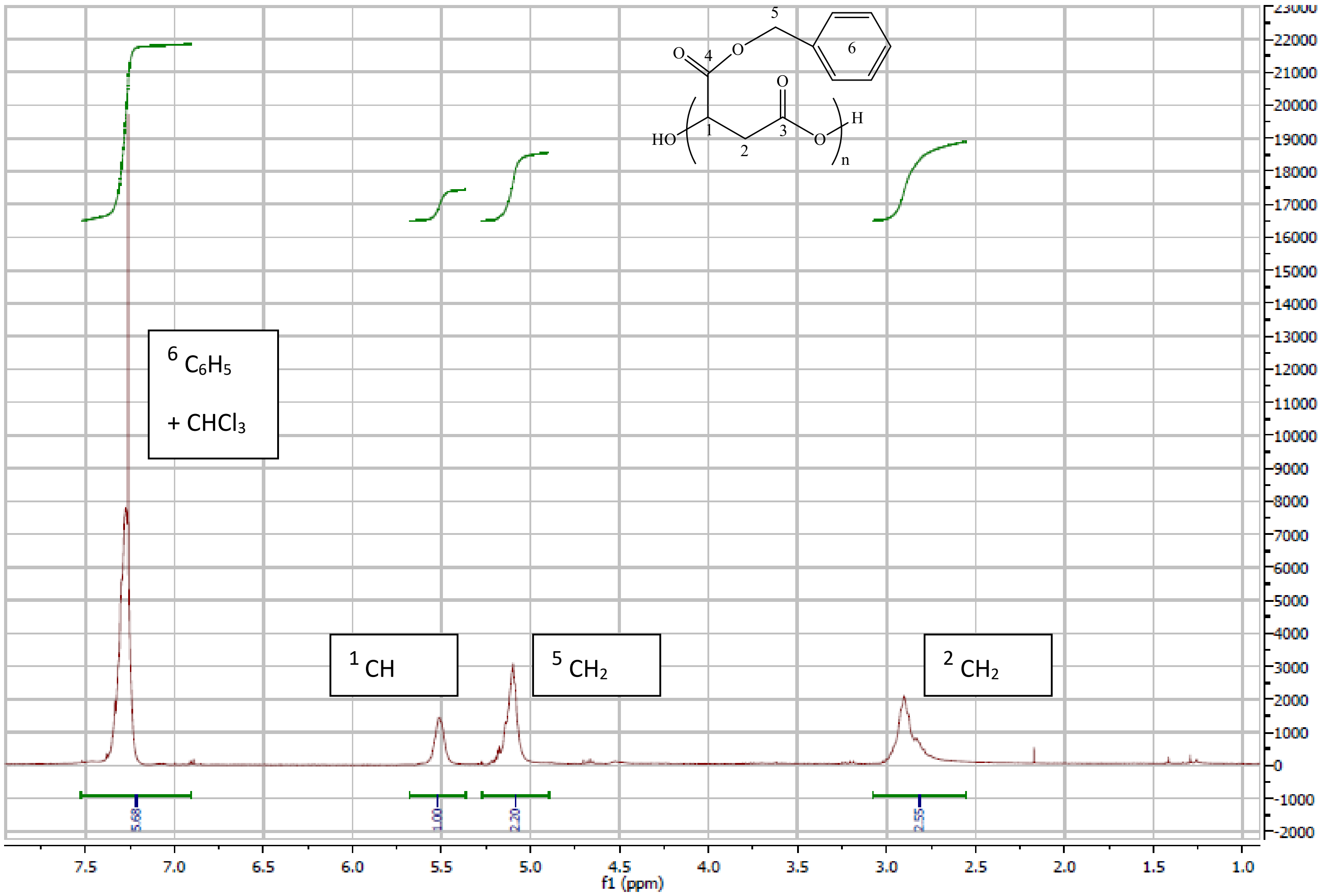

Figure SI 3.8. ^{1}H NMR spectrum of enzymatic PEG$_{17}$-*b*-PMLABe$_{45}$ P8

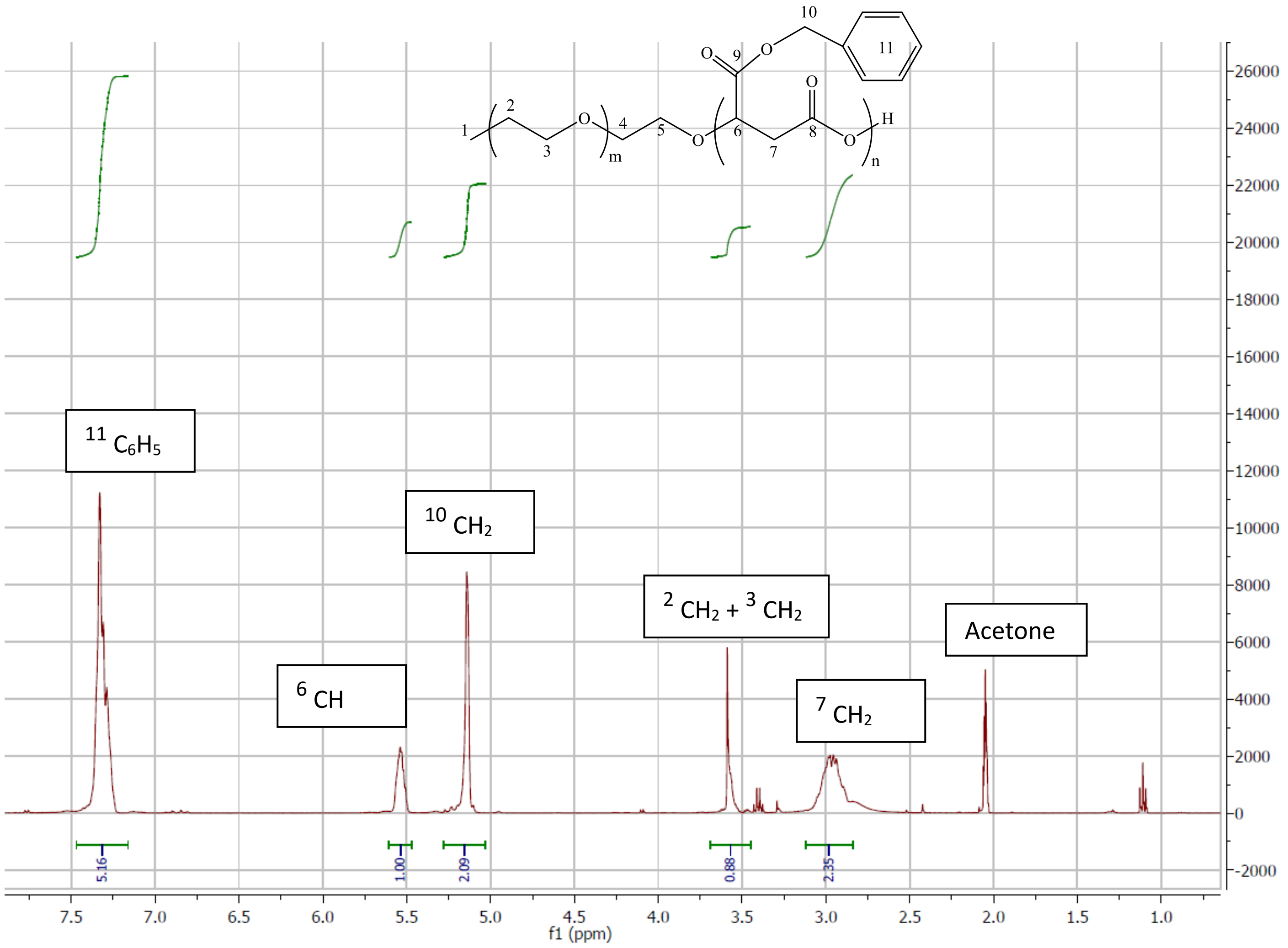

Figure SI 4. *Calibration curve giving the DiR concentration in a DMF/water (80/20) mixture as a function of the Optical Density (DO).*

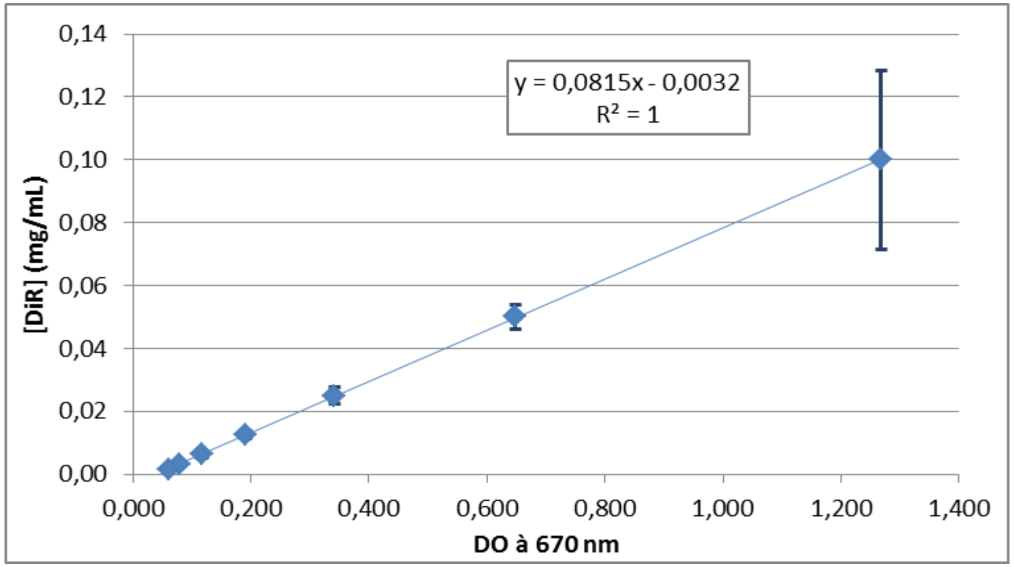

Supplement: Supplementary file 1 [file polymers-10-01244-s001.pdf]
